# Versatile workflow for cell type–resolved transcriptional and epigenetic profiles from cryopreserved human lung

**DOI:** 10.1172/jci.insight.140443

**Published:** 2021-03-22

**Authors:** Maria Llamazares-Prada, Elisa Espinet, Vedrana Mijošek, Uwe Schwartz, Pavlo Lutsik, Raluca Tamas, Mandy Richter, Annika Behrendt, Stephanie T. Pohl, Naja P. Benz, Thomas Muley, Arne Warth, Claus Peter Heußel, Hauke Winter, Jonathan J. M. Landry, Felix J.F. Herth, Tinne C.J. Mertens, Harry Karmouty-Quintana, Ina Koch, Vladimir Benes, Jan O. Korbel, Sebastian M. Waszak, Andreas Trumpp, David M. Wyatt, Heiko F. Stahl, Christoph Plass, Renata Z. Jurkowska

**Affiliations:** 1 BioMed X Institute, Heidelberg, Germany.; 2 Division of Stem Cells and Cancer, German Cancer Research Center (DKFZ), Heidelberg, Germany.; 3 Heidelberg Institute for Stem Cell Technology and Experimental Medicine (HI-STEM), Heidelberg, Germany.; 4 Division of Cancer Epigenomics, DKFZ, Member of the German Center for Lung Research (DZL), Heidelberg, Germany.; 5 Translational Research Unit, Thoraxklinik, University Hospital Heidelberg, Heidelberg, Germany.; 6 Translational Lung Research Center, Member of the DZL, Heidelberg, Germany.; 7 Department of Diagnostic and Interventional Radiology with Nuclear Medicine, Thoraxklinik, University of Heidelberg, Heidelberg, Germany.; 8 Department of Diagnostic and Interventional Radiology, University Hospital Heidelberg, Heidelberg, Germany.; 9 Department of Surgery, Thoraxklinik, University Hospital Heidelberg, Heidelberg, Germany.; 10 Genomics Core Facility, EMBL, Heidelberg, Germany.; 11 Department of Pneumology and Critical Care Medicine and Translational Research Unit, Thoraxklinik, University Hospital Heidelberg, Heidelberg, Germany.; 12 Department of Biochemistry and Molecular Biology, McGovern Medical School, University of Texas Health Science Center at Houston, Houston, USA.; 13 Asklepios Biobank for Lung Diseases, Department of Thoracic Surgery, Asklepios Fachkliniken München-Gauting, DZL, Gauting, Germany.; 14 Genome Biology Unit, EMBL, Heidelberg, Germany.; 15 Biotherapeutics Discovery and; 16 Immunology and Respiratory Disease Research, Boehringer Ingelheim Pharma GmbH & Co. KG, Biberach, Germany.; 17 School of Biosciences, Cardiff University, Cardiff, United Kingdom.

**Keywords:** Pulmonology, Epigenetics, Expression profiling, Lung cancer

## Abstract

Complexity of lung microenvironment and changes in cellular composition during disease make it exceptionally hard to understand molecular mechanisms driving development of chronic lung diseases. Although recent advances in cell type–resolved approaches hold great promise for studying complex diseases, their implementation relies on local access to fresh tissue, as traditional tissue storage methods do not allow viable cell isolation. To overcome these hurdles, we developed a versatile workflow that allows storage of lung tissue with high viability, permits thorough sample quality check before cell isolation, and befits sequencing-based profiling. We demonstrate that cryopreservation enables isolation of multiple cell types from both healthy and diseased lungs. Basal cells from cryopreserved airways retain their differentiation ability, indicating that cellular identity is not altered by cryopreservation. Importantly, using RNA sequencing and EPIC Array, we show that gene expression and DNA methylation signatures are preserved upon cryopreservation, emphasizing the suitability of our workflow for omics profiling of lung cells. Moreover, we obtained high-quality single-cell RNA-sequencing data of cells from cryopreserved human lungs, demonstrating that cryopreservation empowers single-cell approaches. Overall, thanks to its simplicity, our workflow is well suited for prospective tissue collection by academic collaborators and biobanks, opening worldwide access to viable human tissue.

## Graphical abstract

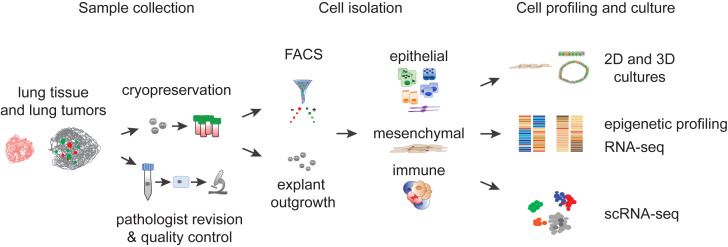

## Introduction

Pulmonary diseases remain among the top 5 causes of death worldwide according to the World Health Organization (1, 2). Lung cancer is the leading cause of cancer-related deaths globally, and despite recent treatment advances, the 5-year survival rate has not improved substantially in the past 20 years (3). Chronic obstructive pulmonary disease (COPD) (4) and idiopathic pulmonary fibrosis (IPF) (5) are devastating lung diseases characterized by progressive airflow limitation and tissue scarring, respectively. To date, limited therapeutic options are available for COPD and IPF; thus, major efforts are made in both academic labs and pharmaceutical industries (4, 6–8) to identify novel drugs targeting key molecular pathways involved in the pathogenesis of these diseases.

Recent advances in next-generation sequencing (NGS) platforms have revolutionized biomedical sciences and the approach to study complex physiological and pathological processes, moving from classical 1-gene studies to a more comprehensive analysis of gene networks (9, 10). NGS-based genetic and epigenetic studies provided the first unbiased maps of lung diseases, leading to the identification of key dysregulated pathways and the discovery of potential disease drivers (11–17). Moreover, the robustness of epigenetic profiling allowed proper classification of cancers of unknown primary origin (18), altogether opening the door for better and personalized treatments. Thus, given the specificity of gene expression and epigenetic profiles, NGS-based profiling strategies hold great promise for biomarker discovery and personalized medicine applications, allowing more precise diagnosis, patient stratification, and even follow-up of patient response to treatment (9, 19–21).

Despite these technological advances, a deep understanding of molecular mechanisms underlying the development of complex lung pathologies is hindered by the heterogeneity of the lung microenvironment (22) and by changes in cellular number and composition during disease progression (15, 17, 23–26), which cannot be resolved with bulk tissue studies. Hence, NGS-based approaches providing cellular resolution, like profiling of defined cell populations or single-cell analyses, need to be implemented to identify cell types driving distinct disease phenotypes. Critically, successful implementation of such experiments is difficult due to shortcomings of human tissue access, tissue quality, and storage platforms.

Foremost, typical lung tissue storage formats offered by biobanks, like flash-frozen or formalin-fixed paraffin-embedded (FFPE) tissue, do not allow viable cell isolation and are therefore not easily compatible with single-cell transcriptome analysis or with epigenetic profiling of defined cell populations. Alternative strategies involving access to fresh tissue via collaborations with local hospitals restrict tissue access geographically. In addition, working with fresh tissue does not allow its thorough histological evaluation before sample processing. Importantly, the quality of clinical samples is crucial for the discovery of disease-relevant changes using NGS-based technologies. For example, the presence of tumor cells, fibrosis, or inflammation in samples selected as controls can significantly increase the experimental noise, masking differential gene expression and epigenetic changes caused by the disease of interest. To circumvent this problem, typically, a very large number of samples must be analyzed, or samples are discarded a posteriori when tissue quality is poor. Both strategies, however, are incompatible with expensive and time-consuming NGS-based experiments, like single-cell RNA-Seq or whole-genome DNA methylation analyses.

Driven by these difficulties existing in the field, we revisited the literature to identify protocols compatible with long-term storage of viable tissue samples (27–31) and established a simplified lung tissue processing workflow that enables tissue collection and expands geographical barriers. We introduced an essential tissue quality check step, where an experienced lung pathologist reviewed the samples before cell isolation and NGS-based profiling. We demonstrate that cryopreservation does not compromise tissue viability and is suitable for isolating multiple cell types from different lung locations. It is applicable to both healthy and diseased lung tissue, including tumors, and it is compatible with NGS-based transcriptional and epigenetic profiling of cells of interest, including single-cell approaches. We also show that cells isolated from cryopreserved human tissue can be used for in vitro validation in cellular models.

## Results

### Lung tissue processing workflow.

**Figure 1:**
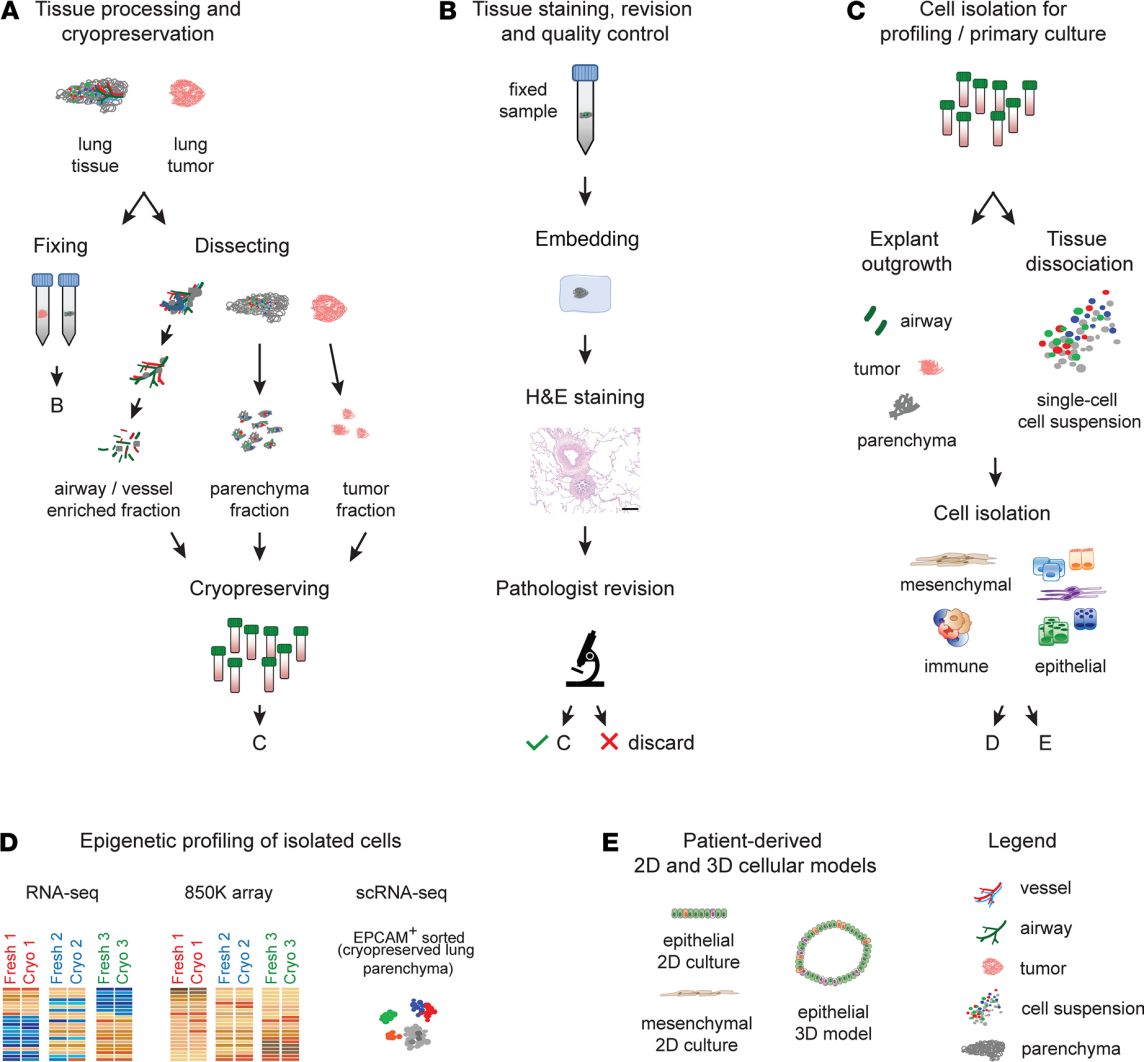
Overview of the tissue processing workflow presented in this study. (**A**) Lung dissection and preparation for histology and cryopreservation of lung tissue and tumor samples. (**B**) Tissue quality control steps, including embedding, H&E staining, and pathological evaluation of the tissue. (**C**) Live cell isolation for profiling and in vitro culture. (**D**) Next-generation sequencing–based profiling, including RNA-Seq, DNA methylation EPIC Array (850K array), and single-cell RNA-Seq (scRNA-Seq). (**E**) Generation of 2D and 3D patient-derived cellular models from cryopreserved tissue. Legend is displayed on the bottom right. EPCAM, epithelial cell-adhesion molecule.

Due to the substantial cost of the NGS-based whole-genome analyses, such as whole-genome bisulfite sequencing or single-cell RNA-Seq, only a limited number of samples are typically analyzed to derive disease signatures. Such setup should be combined with a strict tissue quality analysis to ensure the best possible separation between control and disease groups and the inclusion of high-quality material only. Consequently, we include a cryopreservation step in our experimental workflow to allow a thorough quality check of the tissue samples before performing further downstream assays. Our complete workflow consists of 4 steps: (a) tissue collection and preservation, (b) tissue quality check, (c) cell isolation, and (d) NGS-based profiling (Figure 1). Each step is detailed below.

### Tissue collection and preservation.

First, patient inclusion criteria should be defined for prospective tissue collection studies to ensure the best possible matching of control and disease groups in terms of age, sex, smoking status, and smoking history. In addition, whenever possible, the medical history of the patients should be gathered, for the best possible characterization of the included specimens. This study aimed to evaluate the feasibility and promise of human lung tissue cryopreservation for transcriptomic and epigenetic profiling with cell type resolution. Thus, lung tissue samples (parenchyma from healthy controls, COPD patients, and lung tumors) were collected in transport medium (Supplemental Table 1 and Supplemental Table 11; supplemental material available online with this article; https://doi.org/10.1172/jci.insight.140443DS1) and sent to one of the laboratories involved in the study. At arrival, several representative pieces from different parts of the tissue were collected and fixed in formalin for subsequent histological analysis (Figure 1A and Supplemental Table 2). The remaining material was separated into 3 distinct compartments via manual microscopic dissection, as follows: (1) airway and vessel enriched; (2) airway-free, tumor-free parenchyma; and (3) tumor fraction (Figure 1A). This step was introduced as it allows an easy initial pre-enrichment of different lung compartments, for example, airway versus parenchyma, for subsequent cell isolation. After compartmentalization, the tissue was cut into small sections and cryopreserved for long-term storage (see Methods section for details).

### Cryopreservation enables careful tissue quality control.

**Figure 2:**
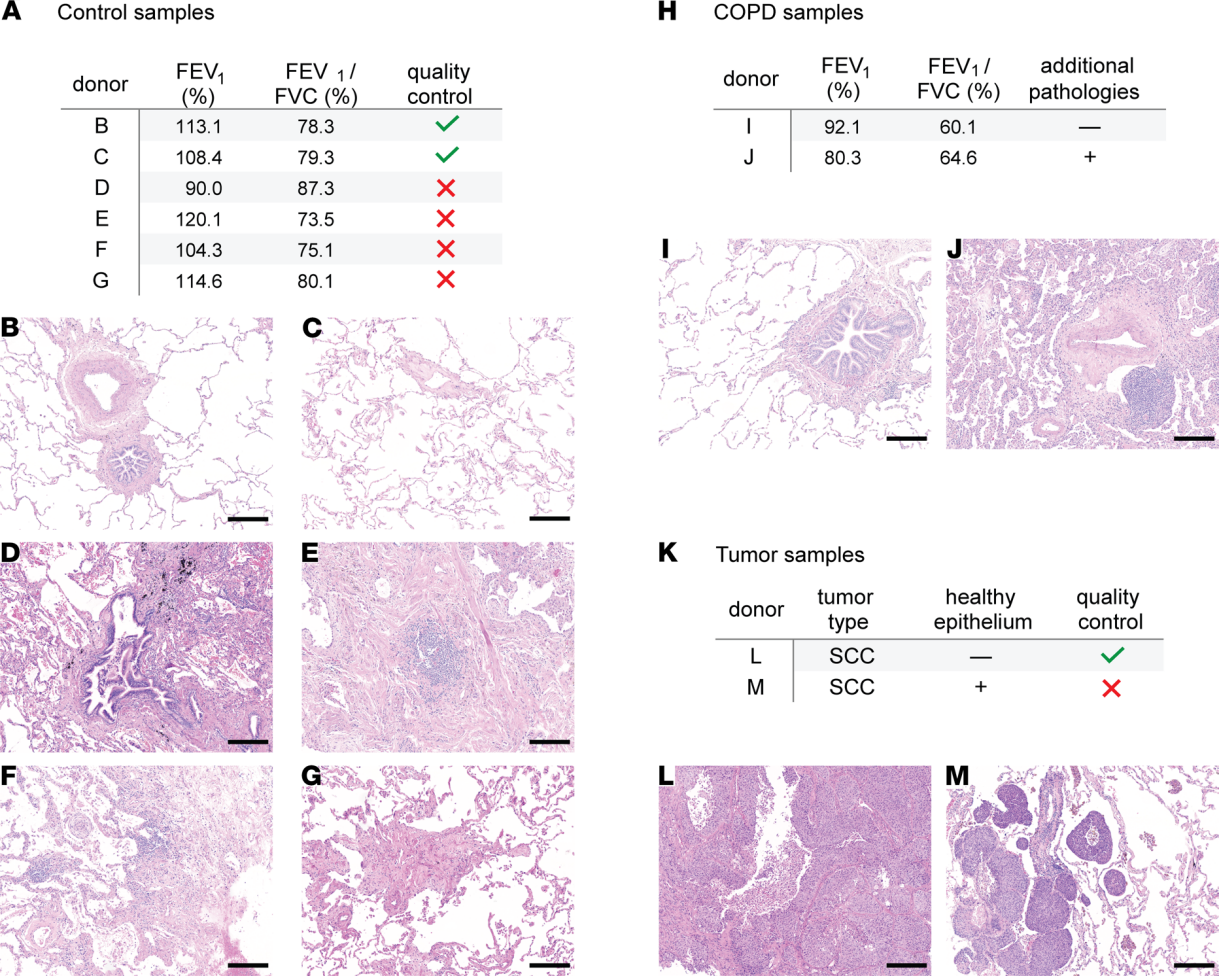
Importance of thorough tissue quality control before cell isolation and profiling. (**A**) Table showing spirometry values (forced expiratory volume in 1 s/forced vital capacity ratio [FEV_1_/FVC] and FEV_1_) of 6 normal donors. (**B**–**G**) Representative H&E images of lung parenchyma from each of the donors listed in the table (**A**). (**B** and **C**) Examples of donors with healthy lungs. (**D**–**G**) H&E images showing slight to moderate fibrosis with mild (**G**) and moderate (**D**–**F**) chronic inflammation and desquamative reaction (**E**). Donor D also presents anthracotic pigment deposits along the bronchovascular bundles. (**H**) Table summarizing the characteristics of 2 exemplary COPD donors. (**I** and **J**) corresponding H&E images showing mild to moderate emphysema (donor I) and moderate fibrosis with thickening of the alveolar walls and chronic inflammation (donor J). (**K**) Table indicating tumor type and presence/absence of healthy epithelium. (**L** and **M**) Exemplary images of 2 lung squamous cell carcinoma (SCC) samples. Tumor L represents a sample with very high tumor purity, whereas tumor M shows the invasion front of a non–small cell lung cancer specimen with intra-alveolar tumor spread (STAS; left side) and a significant amount of healthy lung parenchyma with mild emphysema on the right. Scale bars: 0.2 mm. –, absent; +, present.

Representative tissue slides stained with hematoxylin and eosin (H&E) were carefully evaluated by an experienced lung pathologist for the presence of emphysema, immune infiltrates, fibrosis, as well as other alterations (Figure 1B). This histopathological assessment, together with lung function test results available from clinical records, allows better classification of the collected material. Critically, this step enables the exclusion of low-quality samples, which is essential for the discovery of disease-relevant changes. Remarkably, out of 6 tested “control” samples from donors with preserved lung function and normal radiographic analysis (Figure 2A), only 2 (33%) showed normal histological patterns (Figure 2, B and C). The other 4 tissue samples showed moderate fibrosis with different grades of chronic inflammation (Figure 2, D–G). Notably, all 6 “control” patients had shown normal spirometry values as measured by FEV_1_/FVC and FEV_1_ value close to 100%, demonstrating the importance of the histological quality check (Figure 2A). Similarly, when evaluating diseased samples from patients with COPD (Figure 2, H–J), 1 showed moderate fibrosis with thickening of the alveolar walls and chronic inflammation (Figure 2J). For the tumor tissues (Figure 2, K–M), 1 of the samples contained a considerable amount of normal adjacent tissue (Figure 2M). For samples with high healthy epithelial content, preisolation of tumor cells might be essential to identify tumor-specific signatures and prevent overgrowth of the healthy tissue for functional organoid assays (32–34). In summary, these observations demonstrate that careful histological evaluation of the samples is a critical step before undergoing lengthy cell isolation procedures and expensive downstream sequencing. Selecting proper control and diseased samples based not only on available patient medical data but also on tissue quality will result in more meaningful and unambiguous results during data analysis.

### Cryopreservation does not compromise cell viability.

**Figure 3:**
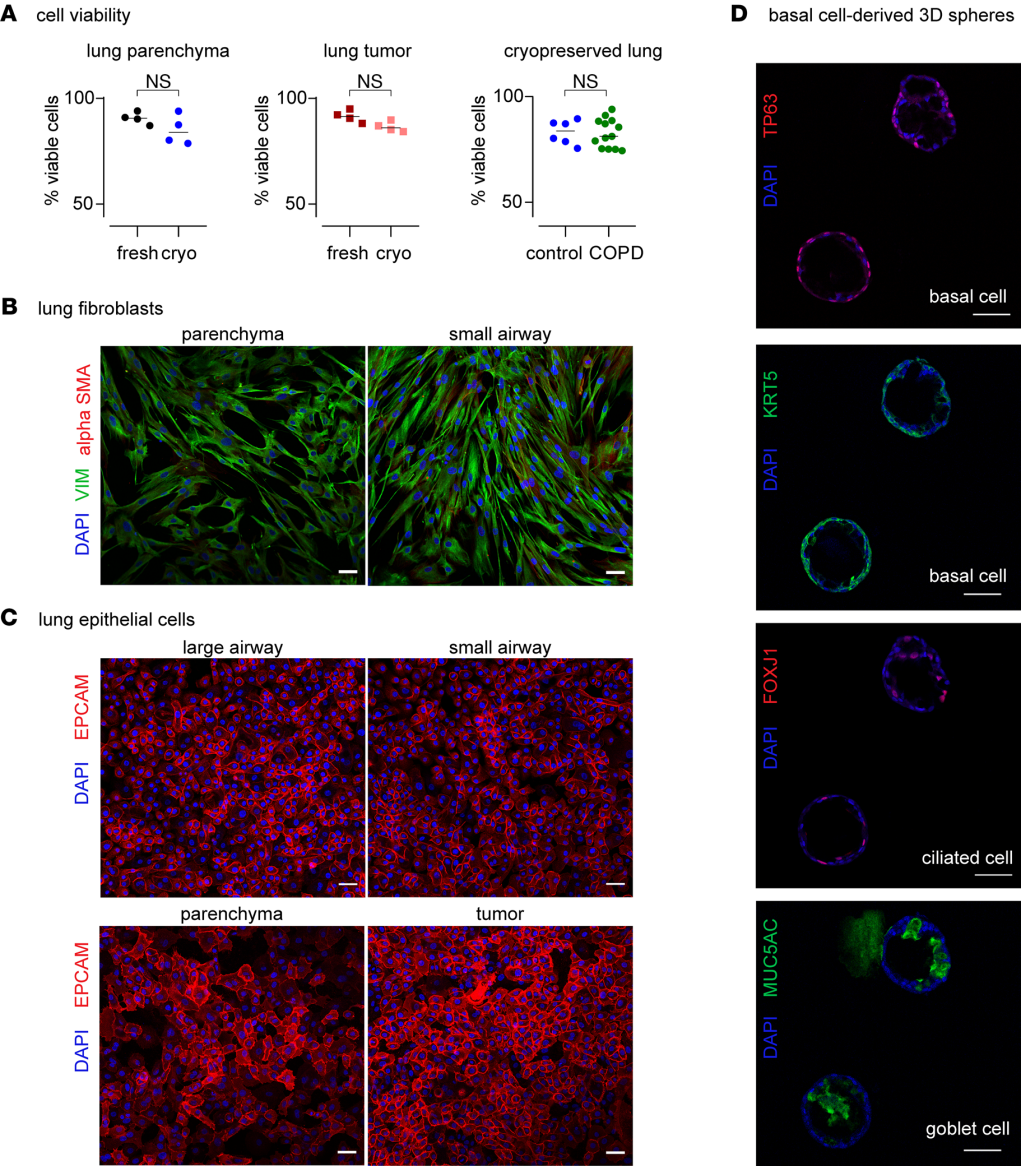
Cell viability and function are maintained in cryopreserved lung samples. (**A**) FACS quantification of viable cells from dissociated lung tissues using SyTOX blue as a viability dye. Comparison between fresh and cryopreserved lung parenchyma (left) and lung tumor (middle) from 4 donors showing overall viability values above 80% (NS, nonsignificant, *P* value= 0.125, *n* = 4, Wilcoxon’s matched pairs signed-rank test). Right, the viability of single-cell suspensions obtained from cryopreserved tissue of healthy control donors (*n* = 6) and COPD (*n* = 13) samples (NS, *P* value = 0.865, Mann-Whitney test). (**B**) Immunofluorescence images of fibroblasts derived from cryopreserved lung parenchyma (left) and small airway (right), demonstrating expression of a mesenchymal marker (vimentin, green). Discrete fibroblasts expressing α–smooth muscle actin (red) are also present. (**C**) Immunofluorescence images of different epithelial cells isolated from cryopreserved material including basal cells from large (top left panel) and small airways (top right panel), distal epithelial cells from parenchyma (bottom left), and tumor epithelial cells derived from lung SCC showing expression of the epithelial marker EPCAM (red). (**D**) Basal cell–derived spheres show that basal cells (TP63^+^, red; KRT5^+^ green, as indicated on the panels) derived from cryopreserved airways are functional and can differentiate into goblet (MUC5AC^+^, green) and ciliated cells (FOXJ1^+^, red). (**B**–**D**) All the nuclei were counterstained with DAPI (blue); scale bars: 50 μm.

To analyze the viability of the cryopreserved lung tissue, tumor-free lung parenchyma as well as tumor samples obtained from 4 donors were processed as follows. First, samples were halved, and one part was cryopreserved. The other half of the tissue was dissociated to generate a single-cell suspension. Cell viability of the total suspension, as well as of the epithelial and immune fractions specifically, was evaluated by flow cytometry using SyTOX staining. Similarly, after 1- or 2-week storage, cryopreserved samples were thawed, dissociated, and processed for comparison with the fresh tissue obtained from the same donor. Although we observed a mild (5% to 10%) viability drop after cryopreservation (Figure 3A and Supplemental Figure 1, A–G), with median viability of 90% for fresh parenchyma (viability range 86%–94%) and of 84% for cryopreserved samples (78%–94%), the differences were nonsignificant (*n* = 4, *P* = 0.125, nonparametric paired Wilcoxon’s test), and both fresh and frozen samples showed high viability with values above 80%. Similar results were obtained for the tumor samples (Figure 3A, with 88%–95% viability for fresh tumor and 85%–90% for cryopreserved tumor), where no significant differences (*n* = 4, *P* = 0.125) in viability were obtained in the total tissue or specific epithelial or immune cell compartments (Supplemental Figure 1, B, C, and E). Moreover, we successfully cryopreserved tissue from smokers with preserved lung function (*n* = 6, viability average = 83.2%), as well as smokers with various stages of COPD (*n* = 13, viability average = 82.7%) (Figure 3A), maintaining viability above 80%, indicating that this protocol can be used to profile both normal and COPD tissue. We, therefore, conclude that the cryopreservation protocol used here preserves tissue viability, and thus, is suitable for isolating viable cells from cryopreserved material.

### Multiple cell types can be isolated from cryopreserved lung tissue.

To assess the compatibility of cryopreservation with the isolation of defined cell populations, which could further be subjected to NGS-based profiling or used for validation studies in vitro, we isolated different mesenchymal and epithelial cell populations from cryopreserved lung tissues as described below.

First, human lung fibroblasts were successfully derived by explant outgrowth from both parenchyma and airway-enriched lung compartments, yielding parenchymal and peribronchial human lung fibroblast populations, respectively (Figure 3B). Immunostaining with antibodies against mesenchymal markers (vimentin and α–smooth muscle actin), complemented with FACS analysis of epithelial (EPCAM) and immune (CD45) markers (Figure 3B and Supplemental Figure 1H), demonstrated high fibroblast purity, indicating that tissue compartmentalization and cryopreservation are compatible with fibroblast isolation from different lung locations. Importantly, as peribronchial and parenchymal fibroblasts show distinct phenotypes (35–37), protocols enabling lung compartmentalization before cell isolation are crucial for understanding specific roles of different fibroblast populations in the development of respiratory diseases.

Second, epithelial cells from cryopreserved airways, parenchymal tissue, and tumor fractions were purified using different protocols (Figure 3C). Human basal epithelial cells and distal alveolar epithelial cells were successfully isolated by explant outgrowth from cryopreserved large and small airways or parenchyma, respectively (Figure 3C and Supplemental Figure 1I, sprout). In addition, parenchyma-derived alveolar epithelial cells were obtained upon thawing, tissue dissociation, and cell seeding on culture dishes or by FACS gating for EPCAM^+^/CD45 cells (Supplemental Figure 1I). Tumor cells were isolated from cryopreserved lung squamous cell carcinomas (SCCs) upon dissociation and seeding (Figure 3C). The purity of the epithelial cells was shown by immunofluorescence and FACS (Figure 3C; Supplemental Figure 1, H and I; and Supplemental Figure 3B), using cell type–specific markers.

Overall, these results show that the cryopreservation protocol described here permits successful isolation and culture of cells from defined lung compartments (airways, parenchyma, and tumors), encompassing different lineages (mesenchymal and epithelial) using alternative isolation methods (FACS, explant outgrowth, or differential seeding after tissue dissociation).

### Cellular identity and functions are not altered because of cryopreservation.

Basal cells are the epithelial progenitors of the human airways (38, 39), which, under specific conditions, can differentiate into ciliated and secretory cells. To evaluate the impact of cryopreservation on the progenitor capabilities of basal epithelial cells, we used a well-established 3D model to generate bronchospheres (40–42). When cultured in ultra-low attachment plates with 5% Matrigel, basal cells derived from cryopreserved airways recapitulated the pseudostratified epithelium observed in vivo as shown by the expression of basal (tumor protein p63 [TP63], keratin 5 [KRT5]), goblet (mucin 5AC [MUC5AC]), and ciliated (forkhead box J1 [FOXJ1]) cell markers (Figure 3D).

These results indicate that lung tissue cryopreservation does not interfere with the isolation of basal cells or with their progenitor capacity. Notably, as the 3D sphere system allows high-throughput studies to model growth, repair, and airway cell differentiation (40), our protocol could facilitate the development of well-characterized patient-derived cellular assays for in vitro studies.

### Cryopreservation preserves transcriptional and epigenetic signatures of cells.

**Figure 4:**
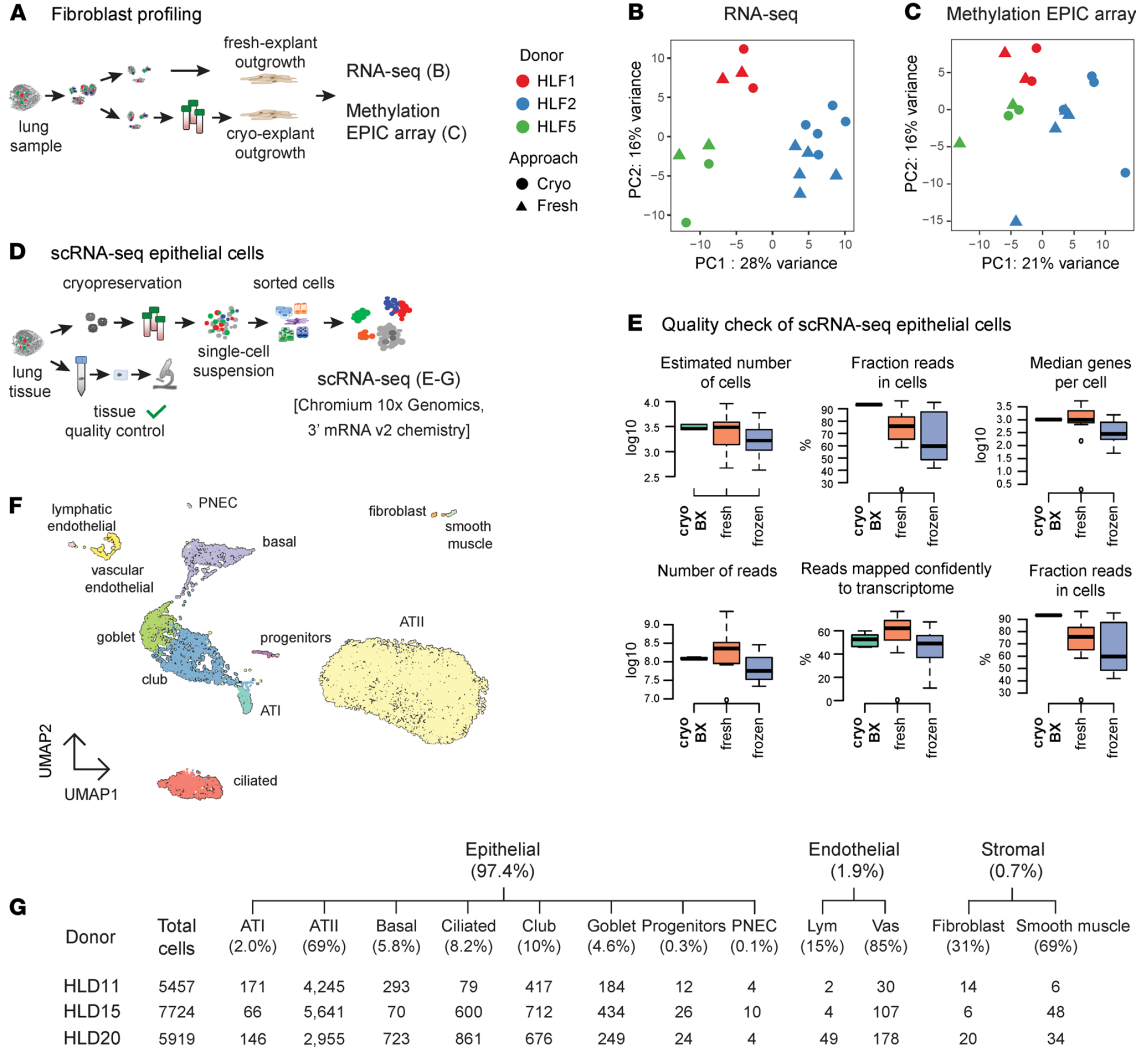
Transcriptional and epigenetic profiles of cells are maintained in cryopreserved lung tissue. (**A**) Overview of the approaches used for comparing genome-wide transcriptional and epigenetic profiles of primary human lung fibroblasts derived from fresh and cryopreserved lung tissue explants of 3 donors. (**B**) Principal component analysis (PCA) of the 500 most variable expressed genes obtained from RNA-Seq across all samples. (**C**) PCA of the 5000 most variable CpG positions revealed by methylation profiling. (**D**) Schematic overview of tissue processing for scRNA-Seq of sorted epithelial cells obtained from cryopreserved parenchyma of 3 control donors. (**E**) Representative quality reports comparing scRNA-Seq data obtained in this study (cryo-BX, green box plot, *n* = 3) with 47 publicly available human data sets from fresh (orange box plot, *n* = 11) and frozen tissue (blue box plot, *n* = 36). The box plots depict the minimum and maximum values (whiskers), the upper and lower quartiles, and the median. The length of the box represents the interquartile range. (**F**) Uniform Manifold Approximation and Projection (UMAP) clustering indicating the main epithelial cell types identified in the epithelial cell–enriched sorted lung parenchyma, including alveolar type I (ATI) and type II (ATII), ciliated, goblet, club, basal, as well as alveolar progenitor cells. Besides the indicated epithelial cells, fibroblasts, smooth muscle cells, pulmonary neuroendocrine cells (PNECs), as well as vascular endothelial and lymphatic endothelial cells, were also identified. Fibroblasts and smooth muscle cells were brought closer for aesthetic reasons. (**G**) Total number of identified cells as well as the averaged percentage and the total number of the different cell populations identified by scRNA-Seq in each of the profiled patients, indicating high reproducibility of the protocol. Vas, vascular endothelial cells; Lym, lymphatic endothelial cells.

To directly evaluate the impact of tissue cryopreservation on the molecular signatures of isolated cells, we obtained genome-wide transcriptional and epigenetic profiles of primary human lung fibroblasts derived by explant outgrowth from fresh and cryopreserved lung parenchyma. For this, lung tissue pieces of 3 independent donors were halved; one part was used to isolate fibroblasts from fresh parenchyma, and the other half underwent cryopreservation before cell isolation (Figure 4A). Importantly, 2 to 5 areas from each patient were included as technical replicates to cover the heterogeneity of the human lung parenchyma.

We consistently obtained good yield and quality of the isolated RNA, as indicated by the RNA integrity number (RIN) higher than 8.0 for all samples. To minimize potential technical batch artifacts in the RNA-Seq experiment, RNA isolation, library preparation, and sequencing of all samples were performed simultaneously. All samples exhibited equally high alignment rates and could be efficiently assigned to the reference gene annotation (alignment statistics are shown in Supplemental Table 9; read count table is provided as Supplemental Table 12), indicative of high-quality RNA-Seq data. Notably, principal component analysis (PCA) of the 500 most variable genes revealed that transcriptome differences between donors represented the highest variance in the data (Figure 4B), and thus, samples derived from the same donor grouped when using unsupervised hierarchical clustering (Supplemental Figure 2C). Critically, a thorough inspection of the first 10 principal components, representing 90% of the total variance in the data, did not reveal a separation of cryopreserved and fresh samples (Supplemental Figure 2, A and B). In summary, the RNA-Seq results indicate that transcriptional signatures of the fresh and cryopreserved samples are highly similar, and no strong gene expression signatures associated with the tissue cryopreservation treatment can be identified.

In parallel, to investigate possible effects of cryopreservation on epigenetic modifications, we performed genome-wide DNA methylation profiling using Human Infinium MethylationEPIC Array on an analogous set of fibroblast samples isolated from fresh and cryopreserved material of the same donors. Quality check, filtering, and normalization of methylation data were performed with the RnBeads pipeline (43) as described in Methods. All samples had a good separation of foreground and background signal according to the internal control probes. Global Euclidean distance-based clustering analysis of data did not reveal a clear cluster structure, with fresh and cryopreserved samples of different donors evenly interspersed (Supplemental Figure 2F). The PCA using the data of all high-quality CpG positions revealed that the major variability was related to donor-specific DNA methylation effects. As for the RNA-Seq data, there was no separation between cryopreserved and fresh samples in any of the planes spanned by pairs of principal components (Figure 4C and Supplemental Figure 2, D and E). The individual-specific variation was stronger than other effects, including cryopreservation, as can be seen in the clustering heatmap of the 5000 most variable CpG positions (Supplemental Figure 2F). Finally, differential methylation analysis for association with the cryopreservation indicator variable did not detect any significantly altered CpG sites after adjustment for multiple testing (under FDR of 0.05), allowing us to conclude that there are no major effects of cryopreservation on the genome-wide scale in DNA methylation profiles. Altogether, these results emphasize the suitability of cryopreservation for the cell type–resolved transcriptional and epigenetic profiling of lung cells.

### Tissue cryopreservation enables high-quality single-cell transcriptomic analysis of isolated lung cells.

Recent advances in single-cell omics profiling have revolutionized biomedical research, allowing identification of novel cell types (44–46), reconstruction of developmental trajectories (47), and fine-mapping of disease states (15–17, 23–26, 48), holding great promise for biomarker discovery and personalized medicine applications. However, as they rely on very high cell viability and integrity, single-cell profiling studies typically require access to fresh tissue, restricting complex experimental designs.

Encouraged by the high viability of our cryopreserved lung tissue, we evaluated the possibility of using our workflow for single-cell transcriptomics. For this aim, we dissociated cryopreserved lung parenchyma of 3 control donors with normal lung function and morphologically healthy lung (based on pathologist evaluation); 2 of them (HLD11 and HLD15) were stored in liquid nitrogen for 2 years. To reduce complexity, we enriched for epithelial cell populations (EPCAM^+^) using FACS and performed single-cell RNA-Seq with the Chromium 10x Genomics technology (Figure 4D and Supplemental Figure 3, A and B). Evaluation of standard single-cell RNA-Seq quality control parameters demonstrated the high quality of the generated single-cell data (Figure 4E and Supplemental Table 10). Importantly, a comparison between our data (cryo-BX, *n* = 3) and 47 publicly available human data sets (fresh, *n* = 11; frozen, *n* = 36) that used the same 10x Genomics 3′ mRNA v2 chemistry revealed that the quality of our data significantly outperformed data obtained from frozen samples and was similar to data obtained from fresh samples (Figure 4E and Supplemental Table 10). Unbiased clustering performed on all cryo-BX samples and 19,100 cells led to the robust identification of 12 distinct cell populations (Figure 4F and Supplemental Tables 13 and 14). Cell clusters were annotated based on commonly used markers in the literature, as well as genes that were differentially expressed among cell clusters (Supplemental Figure 3C and Supplemental Table 15). We identified all major epithelial cell populations in the distal lung: ATII, ATI, basal, ciliated, and secretory cells. In addition, we identified rare cell populations such as PNECs and putative progenitor cells that express ATI, ATII, as well as proliferation markers. Apart from epithelial cells, which constituted 97.4% (*n* = 18,602) of all cells, we also captured 2 endothelial (1.9%, *n* = 370) and 2 stromal (0.7%, *n* = 128) cell populations. Importantly, all cell populations were detected in all 3 donors (Figure 4G), highlighting the reproducibility of the workflow. These data indicate that our cryopreservation workflow preserved high cell viability and integrity and is, therefore, suitable for droplet-based single-cell transcriptome profiling.

## Discussion

The complexity of the lung microenvironment together with changes in cellular composition occurring during disease progression limits our understanding of molecular mechanisms leading to the development of chronic lung diseases and the identification of cell types and changes driving disease phenotypes. Although recent advances in cell type–resolved NGS approaches and single-cell profiling hold great potential for deconvolution of complex disease traits, their implementation greatly relies on local access to fresh tissue. Similarly, the establishment of disease-relevant models based on well-characterized patient-derived primary cells requires a regular supply of fresh lung tissue. Therefore, local tissue access is one of the main limitations for the implementation of research projects based on human samples that have high translational potential.

To overcome this hurdle, we developed and validated a versatile workflow that allows long-term storage of lung tissue samples without compromising cell viability. Our protocol permits thorough histological characterization of lung specimens before cell isolation and enables cell type–resolved transcriptional and epigenetic sequencing-based profiling of human lung samples (Table 1). Importantly, our protocol, compared with more complex methods published previously, like cryopreservation of hPCLS (28), PDX-CP (27), or cryopreservation of digested lung cell suspensions (49), is much simpler. The overall tissue processing does not require any specialized equipment, can be carried out in any laboratory equipped with a cell culture room, and typically requires less than 1 hour for 4 g of lung tissue. Therefore, due to its simplicity, this versatile protocol can be easily implemented in biobanks and research laboratories, as demonstrated by our successful collaboration with the Asklepios Biobank (Germany) and the research group of Karmouty-Quintana (USA) in the present study. Consequently, our workflow opens worldwide access to viable tissue and facilitates future international collaborations and the recruitment of experts at different site locations. Additionally, disconnecting time and location of the tissue collection from the downstream analysis provides further advantages, such as easier logistics, flexibility with experimental planning, and possibility of complex experimental designs requiring specific equipment, such as FACS or single-cell separation devices.

Critically, our workflow includes a step of detailed characterization of the tissue samples before downstream analysis and is, therefore, particularly recommended for studies employing cell type–resolved NGS-based platforms (like single-cell transcriptomic profiling or whole-genome bisulfite sequencing) that are both time-consuming and expensive, and allow processing of only limited sample numbers. Although the implementation of the strict tissue quality step results in a significant dropout of lung samples before cell isolation, it results in a controlled setup, where control and disease groups are divided not only based on clinical parameters (like spirometry or chest tomography) but also based on histopathology of the same tissue piece that is used for downstream analysis. As shown in this study, this step is particularly important for samples from control (nondiseased) donors, which, despite preserved lung function parameters, often display histopathological changes (e.g., emphysema or severe immune infiltration). This is also the case for tumor samples, where high epithelial content of healthy surrounding tissue would lead to confounding results in downstream analysis. Better sample classification reduces experimental noise, provides clearer disease traits, and consequently allows analyzing smaller sample sizes, significantly lowering sequencing costs.

Using primary human lung fibroblasts as a model, we demonstrate the suitability of our protocol for genome-wide transcriptional and epigenetic profiling of defined cell populations derived from cryopreserved lung tissue. Our results indicate that donor-specific transcriptional and epigenetic patterns are preserved during the cryopreservation of lung samples. Earlier this year, a protocol for biobanking of cryopreserved lung cell suspensions for transcriptomic analysis was developed (49). Our mRNA sequencing of parenchymal lung fibroblasts confirms the results of Chu et al. obtained from FACS-sorted ATII and ATI cells using exome capture technology (49) and further emphasizes the suitability of cryopreservation for bulk RNA-Seq of isolated cell populations. Moreover, we demonstrate that DNA methylation patterns of lung fibroblasts are maintained during cryopreservation, suggesting that our protocol is suitable for the epigenetic analysis of isolated lung cells. To our knowledge, this has not been demonstrated systematically before for lung tissue. Hence, cryopreservation of lung tissue pieces could be an alternative approach to cell suspension cryopreservation for lung tissue biobanking. Notably, cryopreservation enables processing of samples from multiple donors in parallel, minimizing technical biases related to multiple batch processing of fresh samples often observed in NGS-based analysis (50). Similarly, our workflow allows generation of excellent-quality single-cell RNA-Seq data from whole cells based on cryopreserved material and reliable identification of multiple epithelial lung cell types, including fragile cells, like ATI or secretory cells, demonstrating that they can be efficiently isolated from the cryopreserved lung tissue. With the development of single-cell transcriptomic technologies and large collaborative single-cell initiatives, like the Human Cell Atlas Project (51–53), various human tissue-processing approaches have been developed. The majority of single-cell studies of the human lung to date relied on direct processing of the collected fresh lung tissue (15–17, 25, 46, 48, 54). However, direct tissue processing at the clinic is usually not practical, may introduce batch effects, and prevents selection of good-quality tissue based on histological analysis. Alternatively, collected samples can be preserved and stored either as tissue or in a dissociated form as single-cell suspensions. Madissoon et al. benchmarked the short-term cold preservation of 3 human tissues (spleen, esophagus mucosa, and lung) for single-cell RNA-Seq analysis and found little effect of cold ischemic time on the quality of the obtained data within the first 24 hours (55). Additional studies assessed a range of cell fixation methods. Several fixatives, including PFA (56), reversible cross-linker dithiobis(succinimidyl propionate) (57), methanol (58–60), and other stabilizing agents (61) have previously allowed the generation of single-cell RNA-Seq data from dissociated cells and tissues. However, fixation created a detectable 3′ bias compared with fresh cells (57). Using established cell lines and primary human cells, several studies demonstrated that DMSO-based cell cryopreservation does not alter global transcriptional profiles of cells (29, 59, 62), despite reduced viability (29). Few studies also demonstrated the suitability of cryopreservation of human tissues — ovarian carcinoma (29), renal biopsies (30), and synovial tissue (63) — for subsequent single-cell RNA-Seq analysis. We extend these observations to the lung tissue and show that cryopreservation of human lung parenchyma as small tissue pieces is also compatible with downstream processing using the 10x Genomics single-cell profiling platform. Additionally, a recent preprint demonstrated that cryopreservation of tumors as solid tissue or dissociated cell suspensions preserves tumor heterogeneity and complexity and provides high-quality single-cell transcriptome data (64). However, small biases in population proportions might occur in cryopreserved samples, as indicated by a depletion of some epithelial cells after cryopreservation of single-cell suspensions of dissociated mouse kidney (60) or human endometrium biopsies (65). Therefore, careful consideration of the experimental design and consistency across samples for a given tissue is recommended.

As previously shown for other lung cryopreservation protocols (27, 28, 31, 49), we demonstrate that viable epithelial and mesenchymal cells can be successfully isolated from different compartments of cryopreserved lung material. Our workflow could be further expanded to other cell lineages, such as immune cells, since their high viability was also preserved during cryopreservation. Importantly, we show that basal epithelial cells isolated from cryopreserved tissue retain their progenitor function and can differentiate into ciliated and goblet cells, opening the possibility of using our platform to model diseases in vitro. Due to its simplicity, our workflow is particularly suitable for the establishment of larger collections of patient-derived material (e.g., lung biopsies) that could enable generation of patient-derived organoids for personalized drug screening, as it is already done for other organs, like the pancreas, kidney, or intestine (32, 66–69).

Finally, as our tissue processing step involves the separation of distinct lung compartments (airway, vessels, parenchyma, tumor), the isolated cells could be used to study crosstalk between multiple cell types from the same donor or between cells from healthy and diseased donors. Such strategies would allow the development of more complex and relevant disease models for drug screening and facilitate investigation of interactions occurring in the lung microenvironment in both healthy and disease states.

In summary, with this simple yet powerful workflow, we hope to encourage prospective collections of well-characterized human lung tissue samples that could be used for cell type–resolved NGS-based profiling and disease modeling using primary human cells, boosting future basic and translational research.

## Methods

### Patient samples.

Specimens were obtained from the Thoraxklinik Heidelberg (Heidelberg, Germany), the Asklepios Biobank (Gauting, Germany), and the University of Texas Health Science Center (Houston, USA). Human tissue samples (tumor or lung parenchyma) were obtained from patients undergoing lung surgery due to primary lung SCC or adenocarcinoma who had not received chemotherapy or radiation within 4 years before surgery. Normal human lung tissue was obtained from the International Institute for the Advancement of Medicine, from lungs rejected for transplantation for reasons unrelated to obvious acute or chronic pulmonary disease.

### Emphysema score index determination.

Lung and emphysema segmentation were performed by calculating the emphysema score index (ESI) from clinically indicated preoperative CT scans taken with mixed technical parameters. After automated lung segmentation using the yet another CT analyzer (YACTA) software, a threshold of –950 Hounsfield units (HU) was used with a noise correction range between –910 and –950 HU to calculate the relative amount of emphysema in % of the respective lung portion (70). While usually global ESI was measured, the contralateral nonaffected lung side was used if a lung was severely affected by the tumor.

*FFPE and H*&*E*. Representative slices from different areas of the tissue and tumor samples were fixed overnight (O/N) with 10% neutral buffered formalin (MilliporeSigma). Next, fixed tissue samples were washed with PBS and kept in 70% ethanol at 4°C. Sample dehydration, paraffin-embedding, and H&E staining were performed at Morphisto GmbH (Frankfurt, Germany). Per sample, 2 sections, 4 μm thick, were cut on a Leica RM2255 microtome with an integrated cooling station and water basin and transferred to adhesive glass slides (Superfrost Plus, Thermo Fisher Scientific). Subsequently, the sections were dried O/N in a 40°C oven to remove excess water and enhance adhesion (see Supplemental Table 2 for details). H&E-stained slides were evaluated by an experienced lung pathologist at the Thoraxklinik in Heidelberg.

### Cryopreservation of primary SCC tumor samples.

Freshly excised lung tumors were transported in CO_2_-independent medium supplemented with 1% BSA, penicillin/streptomycin, and amphotericin (CO_2_-i^+++^, see Supplemental Table 1 for details); washed with cold HBSS; and minced with a sterile razor blade and eye scissors into 4 × 4 mm pieces. Then 10–15 tissue pieces were transferred to cryotubes (Sarstedt) and covered with 1.4 mL ice-cold freezing medium (DMEM supplemented with 10% DMSO and 20% FBS), flipped to distribute the medium within the tissue pieces, and kept on ice for 15 minutes. Tubes were placed in Mr. Frosty containers (Thermo Fisher Scientific) and transferred to –80°C to ensure a cooling rate (1°C/min). For long-term storage, samples were kept in liquid nitrogen.

### Cryopreservation of lung parenchyma and airway- and vessel-enriched fraction.

Lung specimens were transported in CO_2_-i^+++^ (Supplemental Table 1). Tissue pieces were carefully inflated with cold HBSS^++++^ (Supplemental Table 1), and exemplary samples of the different areas of the lung piece were collected for histological analysis (see above). The pleura was carefully removed from the remaining tissue and airways and vessels separated from the parenchyma as much as possible and cryopreserved separately. The parenchymal airway- and vessel-free fraction was further minced with a sterile razor blade and eye scissors into 4 × 4 mm pieces. Then 10–15 pieces were transferred to cryotubes, covered with 1.4 mL ice-cold freezing medium (Supplemental Table 1), kept on ice for 15 minutes, and transferred to –80°C in Mr. Frosty containers to ensure a gradual temperature decrease (1°C/min). For long-term storage, samples were kept in liquid nitrogen. For the airway- and vessel-enriched fraction, after mincing, 5 to 6 pieces of tissue were transferred to cryotubes, covered with 1.4 mL ice-cold CryoStor freezing medium (Supplemental Table 1), kept 15 minutes in ice, and transferred to –80°C in Mr. Frosty containers. The next day, tubes were transferred to a liquid nitrogen tank.

### Tissue dissociation, viability check, and FACS.

Cryopreserved lung and tumor tissues were thawed for 2 minutes in a 37°C water bath, collected in 50 mL Falcon tubes (Neolab Migge), and washed with HBSS^++++^ (see Supplemental Table 5 for details). Fresh and thawed tissue samples were minced into smaller pieces before mechanical and enzymatic dissociation. Tissue pieces were dissociated into single-cell suspensions with the human tumor dissociation kit following manufacturer instructions (Miltenyi Biotec) and published protocols (71, 72). Briefly, 1 g of minced tissue was added to a MACS C tube containing 4.5 mL of CO_2_-i^+++^ (Supplemental Table 1), and the enzyme mix from the human tissue dissociation kit (Miltenyi Biotec), consisting of 200 μL enzyme H, 100 μL enzyme R, 50 μL enzyme A, 10 μM ROCK inhibitor (Y-27632), and 100 μL DNase I, was added (Supplemental Table 5). Tubes were tightly closed and introduced into the MACS dissociator for mechanic disruption, and the following recommended program for lung tissue was performed: program h_tumor_01, followed by a 15-minute incubation at 37°C on a rotator; h_tumor_01, plus 15 minutes at 37°C on a rotator; h_tumor_02, and 15 minutes at 37°C on a rotator for a final enzymatic dissociation and a last mechanical shearing using the program h_tumor_02. The samples were pipetted up and down to help with disaggregating. Finally, the enzymatic reaction was stopped by adding 20% FBS (Gibco, Thermo Fisher Scientific), and single cells were collected by sequential filtering through 100 μm, 70 μm, and 40 μm cell strainers (BD Falcon). Cells were centrifuged 8 minutes at 4°C and 200*g*, resuspended in ACK lysis buffer (MilliporeSigma), and incubated for 3 minutes at room temperature to lyse erythrocytes. After 2 washes with HBSS^++++^, Fc receptors were blocked with human TruStain FcX (BioLegend, Supplemental Table 6) for 30 minutes on ice. Immune and epithelial cells were labeled using different CD45 and EPCAM antibodies (Supplemental Table 6) for 30 minutes in the dark at 4°C following manufacturer instructions. Stained samples were washed, resuspended in HBSS^++++^, and added to FACS tubes with 40 μm cell strainer caps. To discriminate between live and dead cells, we used SyTOX blue as recommended by the manufacturer (Thermo Fisher Scientific, Supplemental Table 6). We sorted live, single-cell gated, EPCAM^+^ cells using a FACSAria II cell sorter (BD Biosciences). Sorted epithelial cells were used for single-cell RNA-Seq analysis or plated for subsequent culture as indicated below. FlowJo software (Tree Star) was used to analyze the FACS results.

### Fibroblast isolation and expansion.

Primary human lung fibroblasts were isolated by explant outgrowth from fresh or cryopreserved tumor-free tissue derived from distal airway-free lung tissue (parenchymal fibroblasts) or small airways (peribronchial fibroblasts) following published protocols (31, 73–75). Briefly, 6–7 microdissected lung parenchyma or airway pieces were placed per well into 6-well plates, left for 30 minutes at room temperature without medium to improve explant attachment, and carefully covered with 1 mL of DMEM (Thermo Fisher Scientific) supplemented with 2% FBS (Gibco, Thermo Fisher Scientific) (DMEM^+++^, Supplemental Table 3). Explants were left undisturbed for 3–4 days, and afterward the medium was exchanged every 2 days and the outgrowth of fibroblasts from the explants was followed daily. Cells were collected from several explant pieces when reaching 70% confluence to preserve the fibroblast heterogeneity. Possible epithelial contamination was prevented by trypsinizing for shorter periods (3 minutes, 0.05% trypsin with EDTA, Thermo Fisher Scientific) and keeping the cells in DMEM^++^ (Supplemental Table 3). Fibroblasts were used within passages 2–4.

### Basal cell isolation and culture.

Primary human basal cells were isolated by explant outgrowth from cryopreserved explants derived from small (diameter < 2 mm) or large airways (diameter > 2 mm) following published protocols (42, 76–78). Briefly, tubes containing cryopreserved airway-enriched fractions were rapidly thawed in a 37°C water bath, transferred to a 2.5 cm dish containing soak buffer (see Supplemental Table 4 for details of buffers and media composition), and soaked twice for 5 minutes. The soaked airway pieces were washed 3 times with a wash buffer. Airways were microdissected in CO_2_-independent medium to remove the remaining parenchymal tissue around them. Microdissected airway pieces were placed into individual wells of a 24-well plate and left in culture with PneumaCult Ex^+++^ undisturbed for 7 days. Afterward, the medium was switched to PneumaCult Ex^+++^ and exchanged every 2 days. Cells were split at 80% confluence using Lonza’s Reagent Pack Subculture Reagents following the manufacturer’s instructions. Basal cells were pelleted at 180*g* for 5 minutes and seeded for immunofluorescence or expanded for 1–2 passages before seeding for 3D sphere cultures.

### Culture and collection of basal cell–derived 3D spheres.

Basal cell–derived 3D spheres were obtained following published protocols (40, 42). Cells in passage 1 were trypsinized and resuspended (3 × 10^4^ cells/mL) in BEGM* (Lonza, see Supplemental Table 4 for details of media composition) containing 5% growth-factor-reduced Matrigel (Corning). A total of 65 μL of the cell suspension was plated in each well of a nonadherent 96-well plate precoated with 30 μL of a 25% solution of Matrigel (Corning) in BEGM* (Lonza). ROCK inhibitor (5 μm, Y-27632) was added at seeding only, and cultures were fed or treated on days 3, 8, and 14 of culture with 70 μL of BEGM*. On day 21, plates containing 3D spheres were placed on ice. The spheres were washed with ice-cold PBS 1X to remove the medium and Matrigel and then collected into Eppendorf tubes. Cells were incubated for 1 hour at room temperature with 4% PFA (Thermo Fisher Scientific), washed with 1X PBS, and embedded in Histogel (Thermo Fisher Scientific) following the manufacturer’s instructions. Histogel-embedded spheres were processed and embedded in paraffin at Morphisto GmbH as detailed above for the tissue specimens.

### Isolation and culture of primary SCC and distal epithelial cells.

Tumor cells were obtained from SCCs and distal epithelial cells isolated from cryopreserved lung parenchyma by mechanical and enzymatic dissociation of cryopreserved tissue as indicated above. Single-cell suspensions were resuspended in SAGM (Lonza) supplemented with 1% FBS (Gibco, Thermo Fisher Scientific) (see Supplemental Table 5 for details).

Afterward, tumor and distal epithelial cells were obtained by modified previously published protocols (79–82) and used within passages 1–3. Specifically, single-cell suspensions were spun down, resuspended in SAGM supplemented with 1% FBS, and placed for 2 hours into 24-well plates at 37°C. The unattached cells were harvested and seeded in new wells and monitored for epithelial growth. When wells contained contaminating fibroblasts in P0, differential trypsinization was performed to eliminate them. Briefly, cells were washed and incubated for 5 minutes at room temperature with 0.25% trypsin (Lonza’s Reagent Pack Subculture Reagents, Supplemental Table 5). Detached cells (mainly fibroblasts) were discarded. If epithelial cells did not reach 70%–80% confluence, trypsin neutralizing solution (TNS) was added to the wells, incubated for 1 minute, and removed, and fresh medium was added to the well. If epithelial cells reached the desired confluence, fresh 0.25% trypsin was added (Lonza’s Reagent Pack Subculture Reagents, Supplemental Table 5), and plates were further incubated at 37°C for 6 minutes. Reaction was stopped with TNS; cells were spun down and resuspended in SAGM supplemented with 1% FBS.

Epithelial cells were isolated by FACS as indicated in the tissue dissociation section above using EPCAM antibodies and cultured with SAGM supplemented with 1% FBS (Supplemental Tables 5 and 6). Additionally, distal alveolar epithelial cells were successfully isolated by explant outgrowth as previously reported with minor modifications (83). Briefly, cryopreserved lung parenchyma pieces were further minced and placed into individual wells of a 24-well plate and left 30 minutes at room temperature for better attachment to the bottom. A total of 200 μL of the SAGM (Lonza) supplemented with 1% FBS (see Supplemental Table 5 for details) was added to each explant well and the plate left undisturbed for 5 days. Afterward, 500 μL of the SAGM (Lonza) supplemented with 1% FBS (Gibco, Thermo Fisher Scientific) was added to each well and the medium replaced every other day. Explants were monitored and cells split at 80% confluence using the Reagent Pack Subculture Reagents (Lonza) following the manufacturer’s instructions (Supplemental Table 5).

### Immunofluorescence.

Ten thousand human lung fibroblasts derived from cryopreserved explants or 5000 epithelial cells obtained from cryopreserved lung material were seeded per well in a 96-well plate (in passage 3 or passage 1, respectively). After 24 to 48 hours, cells were washed with PBS 1X, fixed for 10 minutes with 4% PFA (MilliporeSigma) at room temperature, and washed and permeabilized for 10 minutes with 0.3% Triton X-100 (Gibco, Thermo Fisher Scientific) at room temperature. Unspecific staining was blocked by incubating 1 hour at room temperature with blocking buffer (for details of buffer composition and antibodies see Supplemental Table 7). Cells were incubated with the indicated primary antibodies overnight at 4°C. Cells were washed with PBS 1× and labeled with respective secondary antibodies for 40 minutes at room temperature in the dark. Finally, cells were washed with PBS 1X, counterstained with DAPI (1:5000, MilliporeSigma) for 10 minutes at room temperature, washed once more with PBS 1X, and kept at 4°C with PBS 1X until imaging.

Slides containing 4 μm thick slices of 3D spheres were deparaffinized and rehydrated as indicated in Supplemental Table 2 (steps 30–38). Heat-induced epitope retrieval was performed in a pH 6.0 citrate buffer (pH 6.0, Abcam) (Supplemental Table 2, step 39) for 20 minutes at 100°C. Slides were washed, permeabilized, and treated as indicated above for immunofluorescence of cells (Supplemental Table 7). Finally, 3D sphere–containing slides were mounted with ProLong Antifade reagent (Thermo Fisher Scientific) (Supplemental Table 7) containing DAPI. Microscope slides were left to dry overnight before imaging.

Imaging of cells and spheres was conducted at the Zentrum für Molekulare Biologie der Universität Heidelberg Imaging Facility (Heidelberg) using the Zeiss LSM780 confocal fluorescence microscope.

### RNA isolation and RNA-Seq.

A total of 2 × 10^5^ primary human lung fibroblasts obtained from fresh and cryopreserved explants from 3 donors in 2–5 technical replicates were harvested at passage 3. Total RNA was isolated using the RNeasy Plus Micro Kit (Qiagen) following the manufacturer’s instructions (for details of reagents please see Supplemental Table 8). DNA was removed by passing the lysate through the genomic DNA eliminator column provided with the kit and by on-column DNase treatment before elution (Qiagen). RNA was eluted using nuclease-free water (Thermo Fisher Scientific) and the concentration measured with Qubit RNA Assay Kit in Qubit 3.0 Fluorometer (Thermo Fisher Scientific). RNA integrity was assessed using the RNA Pico 6000 Assay Kit of the Bioanalyzer 2100 system (Agilent Technologies), and only samples with RIN higher than 8 were processed further. Stranded mRNA-Seq libraries were prepared at the EMBL GeneCore from 200 ng of total RNA using the Illumina TruSeq RNA Sample Prep v2 Kit implemented on the liquid handling robot Beckman FXP2. The libraries were pooled in equimolar amounts, and 1.8 pM solution of each pool was loaded on the Illumina sequencer NextSeq 500 high output and sequenced unidirectionally, generating approximately 450 million reads per run, each 75 bases long.

### Alignment and transcript abundance quantification.

Single-end reads from the RNA-Seq experiment were mapped to the human genome version 37 and the reference gene annotation (release 70, Ensembl) using STAR v2.5.0a (84) with following parameters: --outFilterType BySJout --outFilterMultimapNmax 20 --alignSJoverhangMin 8 --alignSJDBoverhangMin 1 --outFilterMismatchNmax 999 --alignIntronMin 20 --alignIntronMax 100000 --outFilterMismatchNoverReadLmax 0.04 --outSAMtype BAM SortedByCoordinate --outSAMmultNmax 1 --outMultimapperOrder Random.

The contamination of PCR duplication artifacts in the RNA-Seq data was controlled using the R package dupRadar (85). The featureCounts script (86) of the Subread package v1.5.3 was used to assign and count mapped reads to annotated protein-coding and long noncoding RNA genes with default settings.

### Exploratory data analysis.

For exploratory RNA-Seq data analysis, the data needed to be homoscedastic. Therefore, the raw counts were transformed by the regularized-logarithm transformation rlog built in to the DESeq2 Bioconductor package (87). The 500 genes with the highest variance in expression across all samples were subjected to PCA using the R function prcomp and to hierarchical clustering using the pheatmap Bioconductor package (88).

### DNA extraction and Infinium MethylationEPIC BeadChip assay.

Genomic DNA was extracted from 2 × 10^5^ primary human lung fibroblasts isolated from fresh and cryopreserved tissue from 3 donors in technical replicates using QIAamp Micro Kit (Qiagen) following the manufacturer’s protocol, with an additional RNase A treatment step (Qiagen).

Infinium MethylationEPIC BeadChip assay genome-wide DNA methylation profiles of human lung fibroblasts were generated using Illumina’s Infinium MethylationEPIC Bead Chip assay (EPIC Array) at the Genomics and Proteomics Core Facility at DKFZ (Heidelberg, Germany). The assay allows determination of DNA methylation levels at more than 850,000 CpG sites and provides coverage of CpG islands, RefSeq genes, ENCODE open chromatin, ENCODE transcription factor binding sites, and FANTOM5 enhancers. The assay was performed according to the manufacturer’s instructions and scanned on an Illumina HiScan. To avoid batch effects, both fresh and cryopreserved samples from the same donor were assayed on the same array.

### Infinium MethylationEPIC BeadChip data processing.

Primary array data as IDAT files were loaded into R and preprocessed using the package RnBeads (43). In addition, we applied stringent filtering to exclude probes potentially overlapping with common genetic variants (dbSNP v.150) with minor allele higher or equal to 1% within 3 bp from the queried CpG position (PCA, correlation analysis on all probes and heatmaps as in the case of gene expression, with the 5000 most variable sites). Differential methylation analysis was performed using RnBeads. In brief, a linear model with cryopreservation as an independent indicator variable was fit, and the corresponding coefficient was tested for significance using *limma* package (89). Adjustment for multiple testing was performed using Benjamini-Hochberg method (90).

### Single-cell RNA-Seq library preparation.

EPCAM^+^ FACS-sorted cells from 3 donors with normal lung function as determined by spirometry and normal lung morphology confirmed by pathological evaluation were spun down, resuspended in HBSS^++++^ (Supplemental Table 1, Supplemental Table 5, and Supplemental Table 11), and counted. Samples were submitted to the EMBL GeneCore for processing on 10x Genomics Chromium and sequencing. Briefly, 8700 living EPCAM^+^ epithelial cells were loaded into the 10x Genomics Chromium controller using chip A according to the manufacturer’s protocol. Reverse transcription, cDNA amplification, and the subsequent library preparation from 250 ng of cDNA were performed following 10x Genomics Single Cell 3′ gene expression protocol. The finished libraries were pooled and sequenced using Illumina NextSeq 550 instrument with an asymmetric read mode 26 bp read 1 with an 8 bp index read and 58 bp read 2.

### Single-cell RNA-Seq data processing, alignment, clustering, and cell type classification.

The 10x single-cell RNA-Seq data were processed using the Cell Ranger (v3.0.0) analysis pipeline (default parameters). Reads were aligned against the human reference genome GRCh38. The Seurat (v3.0.1) R package was used for downstream data analysis. For each sample, we filtered out cells with unique molecular identifiers < 500, unique molecular identifiers > 2500 (>3 SD from the mean), and mitochondrial mapping percentage > 10%. Normalization for technical confounders and variance stabilization was based on regularized negative binomial regression and the sctransform (v0.2.0) R package with default parameters. The mitochondrial mapping percentage was further regressed out in an additional nonregularized linear regression step. The integration of all 3 lung data sets was based on the Seurat v3 integration workflow (default parameters). A graph-based clustering approach was used to identify cell clusters. Briefly, the first 50 principal components were used to build a K-nearest neighbor graph, and cell clusters were identified using the Louvain algorithm (resolution = 2.0). Visualization of cell clusters was based on a low dimensional projection of the first 50 principal components using UMAP and the umap (v0.2.2) R package (n_neighbors = 30, min_dist = 0.3, metric = cosine). Differentially expressed genes between cell clusters and marker genes were identified using Wilcoxon’s rank-sum tests and the FindAllMarkers Seurat function (logfc.threshold = 0.2, min.pct = 0.2, only.pos = true). Cell clusters were manually annotated and merged into 12 distinct cell types (8 epithelial, 2 endothelial, and 2 stromal) based on commonly used markers in the literature as well as genes that were differentially expressed among cell clusters. We assessed the quality of the cryopreservation protocol for single-cell mRNA sequencing based on comparison with quality control reports (https://10xqc.com) for fresh (*n* = 11) and frozen (*n* = 36) human samples (Chemistry Description = Single Cell 3′ v2, scRNA-Seq method = 10x Genomics 3′ mRNA v2, Transcriptome = GRCh38).

### Data availability.

The bulk RNA-Seq, Illumina EPIC Array DNA methylation, and single-cell RNA-Seq data generated in this study have been deposited at the European Genome-phenome Archive, which is hosted by the European Bioinformatics Institute and the Centre for Genomic Regulation, under accession number EGAS00001004477.

### Statistics.

A paired nonparametric test (Wilcoxon’s matched pairs signed-rank test, GraphPad Prism software, version 8.0.1) was employed to compare the viability between fresh and cryopreserved lung tissue from the same donor. To compare the viability between cryopreserved material from control versus COPD samples, unpaired nonparametric *t* test was employed (Mann-Whitney test). The *P* value of 0.05 was considered significant.

### Study approval.

The protocol for tissue collection was approved by the ethics committees of the University of Heidelberg (S-270/2001), Ludwig-Maximilians-Universität München (projects 333-10 and 17-166), and institutional review board of University of Texas Health Science Center at Houston (HSC-MS-08-0354) and followed the guidelines of the Declaration of Helsinki. All patients gave written informed consent before inclusion in the study and remained anonymous in the context of this study.

## Author contributions

RZJ, MLP, and EE contributed to the design and conception of the study. MLP performed most of the experiments with help from EE, VM, AB, STP, RT, MR, and NPB. TM, FJFH, HW, IK, HKQ, and TCJM provided lung tissue and patient data. AW performed the pathological analysis of the H&E lung specimens. CPH determined the ESI of the patients from whom tissue samples were taken. EE developed the initial tissue cryopreservation protocol. US analyzed the bulk RNA-Seq data, IPL analyzed the EPIC Array DNA methylation data, JJML preprocessed single-cell RNA-Seq data, and SMW performed single-cell gene expression analysis. HFS, AT, VB, JOK, DMW, and CP provided critical input and materials. MLP and RZJ wrote the manuscript with input from all authors. All authors contributed to scientific discussions and approved the final version of the manuscript.

## Supplementary Material

Supplemental data

Supplemental Table 12

Supplemental Table 13

Supplemental Table 14

Supplemental Table 15

## Figures and Tables

**Table 1:**
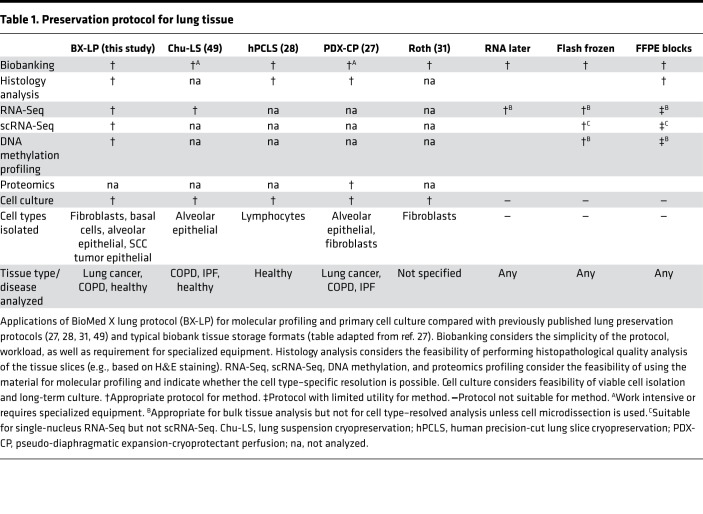
Preservation protocol for lung tissue

## Version 1 02/25/2021

In-Press Preview

## Version 2 03/22/2021

Electronic publication
