# Supplementary material for: Versatile workflow for cell type–resolved transcriptional and epigenetic profiles from cryopreserved human lung
Source: JCI Insight. 2021 Mar 22;6(6):e140443. doi: 10.1172/jci.insight.140443 (PMC8026197; doi:10.1172/jci.insight.140443)
Supplement: Supplemental data [file jciinsight-6-140443-s152.pdf]

Supplementary information and figures

Versatile workflow for cell-type resolved transcriptional and epigenetic profiles from cryopreserved human lung.

Maria Llamazares Prada[1¥], Elisa Espinet[2,3], Vedrana Mijošek[1], Uwe Schwartz[1/], Pavlo Lutsik[4], Raluca Tamas[1], Mandy Richter[1], Annika Behrendt[1], Stephanie T. Pohl [1], Naja P. Benz[1], Thomas Muley[5,6], Arne Warth[5ǁ], Claus Peter Heußel[6,7,8], Hauke Winter[6,9], Jonathan J. M. Landry[10], Felix J.F. Herth[5,11], Tinne C.J. Mertens[12], Harry Karmouty-Quintana[12], Ina Koch[13], Vladimir Benes[10], Jan O. Korbel[14], Sebastian M. Waszak[14^], Andreas Trumpp[2,3] David M. Wyatt[15], Heiko F. Stahl[16], Christoph Plass[4] and Renata Z. Jurkowska[1#*]

[1] BioMed X Innovation Center, Heidelberg, Germany

[2] Division of Stem Cells and Cancer, German Cancer Research Center (DKFZ), Heidelberg, Germany

[3] Heidelberg Institute for Stem Cell Technology and Experimental Medicine (HI-STEM, Heidelberg, Germany

[4] Division of Cancer Epigenomics, German Cancer Research Center (DKFZ), Heidelberg, Germany, Member of the German Center for Lung Research (DZL), Heidelberg, Germany

[5] Translational Research Unit, Thoraxklinik, University Hospital Heidelberg, Germany

[6] Translational Lung Research Center, Member of the German Center for Lung Research (DZL), Heidelberg, Germany

[7] Diagnostic and Interventional Radiology with Nuclear Medicine, Thoraxklinik, University of Heidelberg, Heidelberg, Germany

[8] Diagnostic and Interventional Radiology, University Hospital Heidelberg, Heidelberg, Germany

[9] Dept. of Surgery, Thoraxklinik, University Hospital Heidelberg, Heidelberg, Germany

[10] Genomics Core Facility, European Molecular Biology Laboratory (EMBL), Heidelberg, Germany

[11] Department of Pneumology and Critical Care Medicine and Translational Research Unit, Thoraxklinik, University Hospital Heidelberg, Heidelberg, Germany

[12] Department of Biochemistry and Molecular Biology, McGovern Medical School, University of Texas Health Science Center at Houston, Houston, USA

[13] Asklepios Biobank for Lung Diseases, Department of Thoracic Surgery, Asklepios Fachkliniken München-Gauting, German Center for Lung Research (DZL)f, Gauting, Germany

[14] Genome Biology Unit, European Molecular Biology Laboratory (EMBL), Heidelberg, Germany

[15] Biotherapeutics Discovery, Boehringer Ingelheim Pharma GmbH & Co. KG -Biberach, Germany

[16] Immunology and Respiratory Disease Research, Boehringer Ingelheim Pharma GmbH & Co. KG – Biberach, Germany

[¥] Current address: Division of Cancer Epigenomics, German Cancer Research Center (DKFZ), Heidelberg, Germany, Member of the German Center for Lung Research (DZL), Heidelberg, Germany

[/] Current address: Computational Core Unit, University of Regensburg, 93053 Regensburg, Germany

[‖] Current address: Institute of Pathology, Cytopathology and Molecular Pathology MVZ UEGP Gießen/Wetzlar/Limburg/Bad Hersfeld, Germany

[^] Current address: Centre for Molecular Medicine Norway (NCMM), Nordic EMBL Partnership, University of Oslo and Oslo University Figure Hospital, Oslo, Norway

[#] Current address: Cardiff University, School of Biosciences, Cardiff, Wales, UK.

* Correspondence should be addressed to jurkowskar@cardiff.ac.uk, telephone +44 29 2087 9067 Mailing address: Cardiff University, School of Biosciences, Museum Avenue, CF10 3AX, Cardiff, Wales, UK

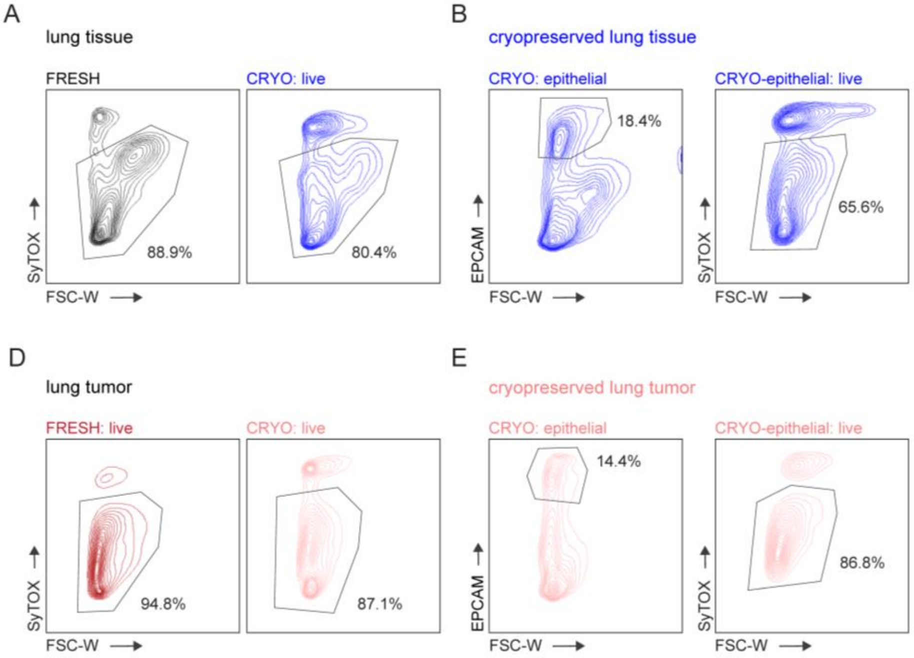

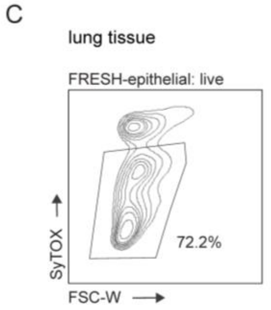

F

SyTOX⁻ viable cells (%)

Patient	Fresh tissue	Cryo tissue
HLD2	90.3	87.6
HLD6	95.7	91.5
HLD8	88.9	80.4
HLD10	86.1	78.2

G

SyTOX⁻ viable cells (%)

Tumor	Tumor type	Fresh tumor	Cryo tumor
T1	SCC	88.2	85.3
T2	SCC	93.7	88.2
T3	ADC	90.5	84.7
T4	ADC	94.8	87.1

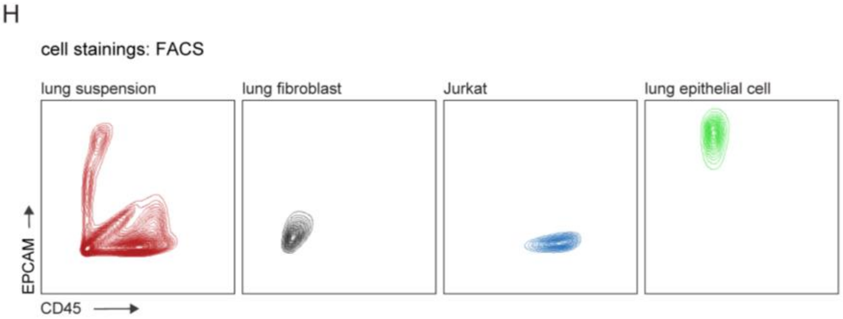

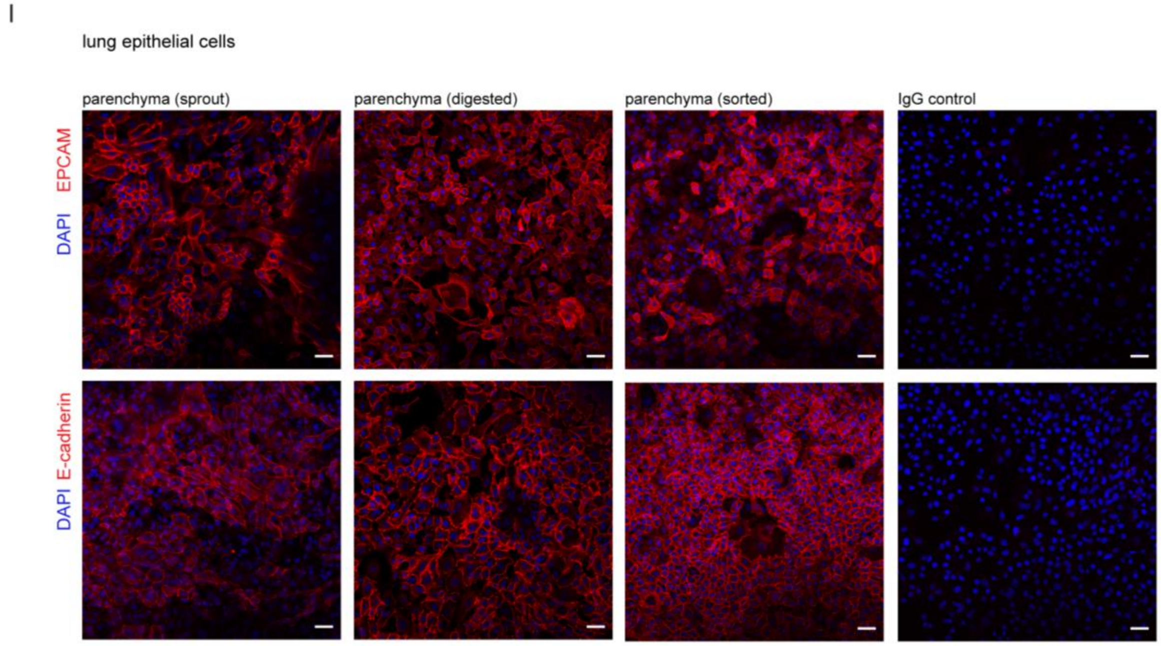

Figure S1. High cell viability is maintained in cryopreserved lung samples allowing live cell isolation. A. Exemplary contour plots obtained by cytometric analysis of a representative donor indicating the gating strategy to determine cell viability (SyTOX$^-$ cells, gated) in single-cell suspensions from fresh (left, black plot, 88.9% viability) and cryopreserved lung samples (right, blue plot, 80.4% viability) of the same donor. **B.** Contour FACS plots showing epithelial cell content (EPCAM$^+$, 18%, left panel) and viability (SyTOX$^-$, 66%) within this population in cryopreserved lung tissue from donor shown in A. **C.** Viability plot from fresh epithelial cells (72%) from the donor shown in A and B. **D.** Representative contour plots indicating the viability of fresh (dark red, left, 94.8%) and cryopreserved lung tumors (light red, 87.1%) from the same donor. **E.** Epithelial content (14.4%) and viability (86.8%) of epithelial cells from the cryopreserved lung tumor shown in D. **F.** Summary table depicting the percentage of viable cells in fresh and cryopreserved lung tissue from 4 different donors measured by FACS and shown in Figure 3A. **G**. Tumor type (ADC, adenocarcinoma; SCC, squamous-cell carcinoma) and percentage of viable cells in fresh and cryopreserved lung tumors from 4 different donors, analyzed by FACS as indicated above and shown in Figure 3A. **H.** FACS plots showing the absence or presence of epithelial (EPCAM$^+$) and hematopoietic (CD45$^+$) markers in lung single-cell suspension (EPCAM$^+$CD45$^-$, EPCAM$^-$CD45$^+$ and EPCAM$^-$CD45$^-$ cells), purified lung fibroblast (EPCAM$^-$CD45$^-$), Jurkat cells (used as a control for CD45$^+$) and epithelial cells isolated from distal lung parenchyma (EPCAM$^+$). **I**. Immunofluorescence staining showing the presence of EPCAM (red, top) and E-cadherin (red, bottom) in the surface of lung epithelial cells obtained by sprouting, dissociation or FACS sorting of cryopreserved lung parenchyma. Right, IgG control. All the nuclei were counterstained with DAPI (blue); scale bars: 50 µM.

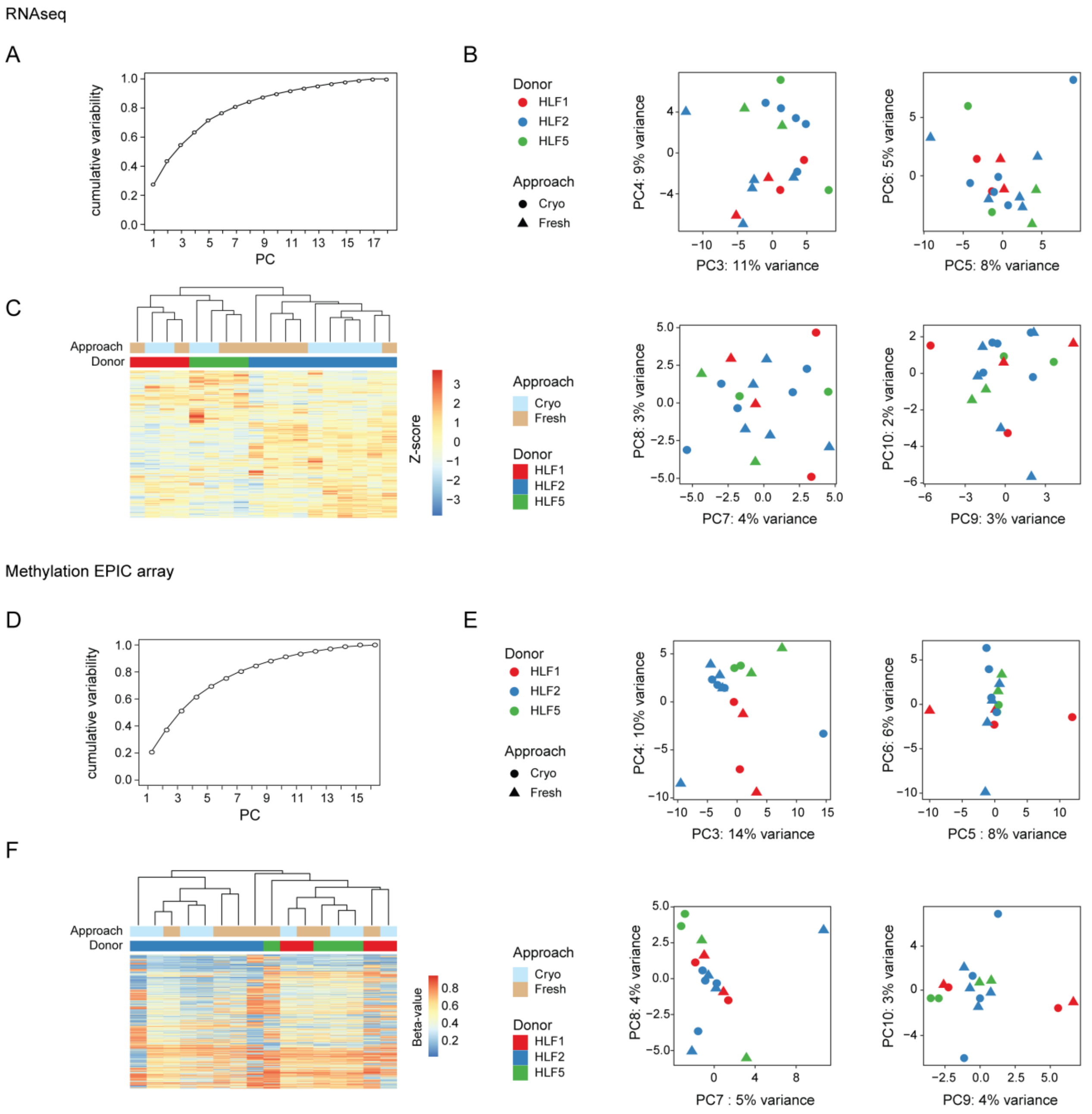

Figure S2. **Quality-control of the RNA-seq (A-C) and Methylation EPIC array data (D-F) obtained from human lung fibroblasts (HLF) isolated from 3 donors. A**. Plot showing the cumulative variance represented in the RNA-seq data by the principal components. **B**. Principal component analysis of the 500 most variable genes across all samples. Principal components 3-10 are shown. The shape of the dots represents the preparation of the sample (circle, cryopreserved; triangle, fresh) and the color code indicates the donor as shown in the legend. **C**. Hierarchical clustering of the samples on the 500 most variable genes. **D**.

Cumulative variance present in the methylation data by the principal components. **E**. Principal component analysis of the 5000 most variable CpG sites across all samples. Principal components 3-10 are shown. **F**. Hierarchical clustering of the samples on the 5000 most variable CpG sites.

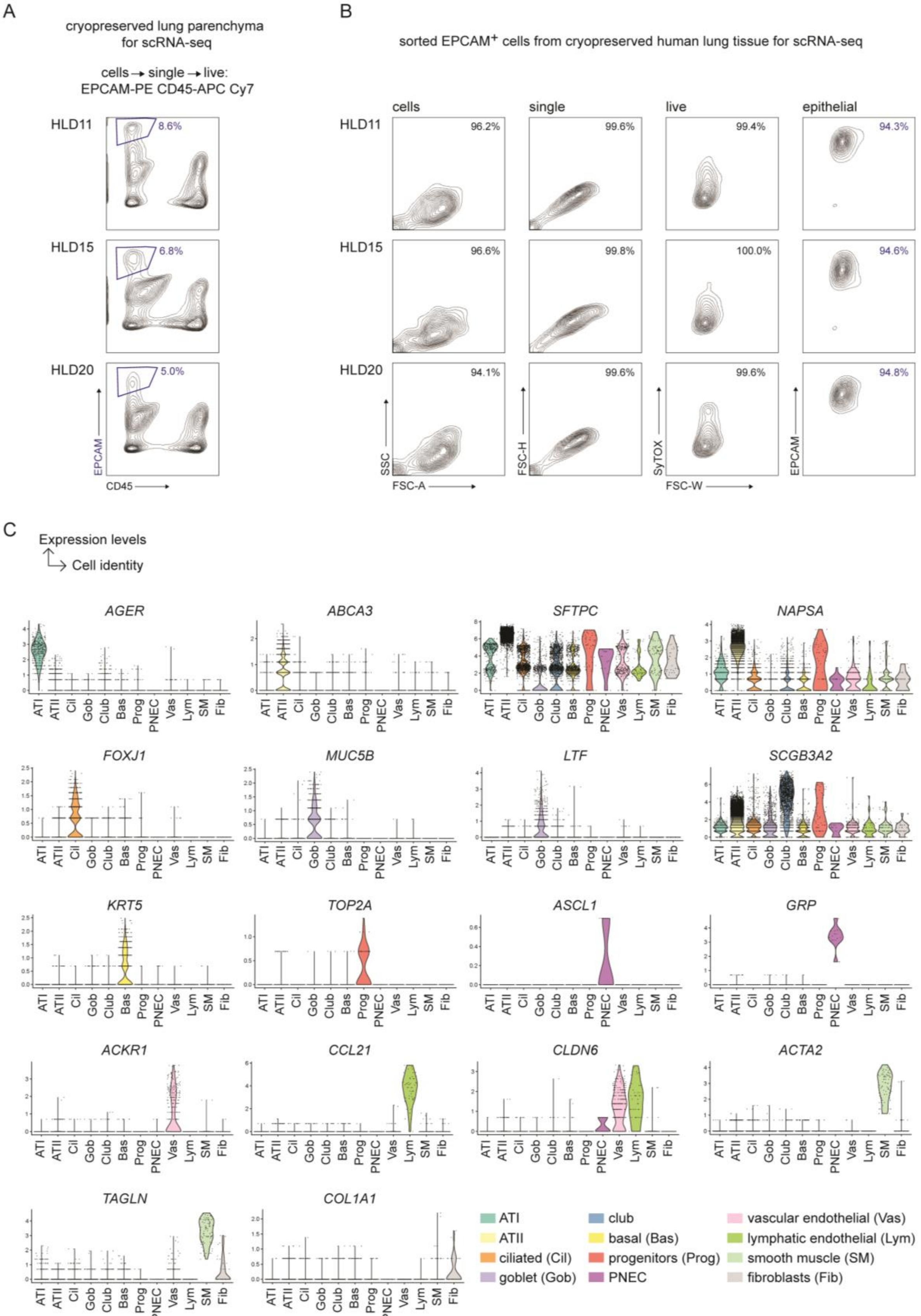
A
cryopreserved lung parenchyma for scRNA-seq
cells → single → live: EPCAM-PE CD45-APC Cy7
HLD11
8.6%
HLD15
6.8%
HLD20
5.0%
EPCAM
CD45
B
sorted EPCAM+ cells from cryopreserved human lung tissue for scRNA-seq
cells
single
live
epithelial
HLD11
96.2%
99.6%
99.4%
94.3%
HLD15
96.6%
99.8%
100.0%
94.6%
HLD20
94.1%
99.6%
99.6%
94.8%
SSC
FSC-A
FSC-H
SyTOX
FSC-W
EPCAM
C
Expression levels
Cell identity
AGER
ABCA3
SFTPC
NAPSA
FOXJ1
MUC5B
LTF
SCGB3A2
KRT5
TOP2A
ASCL1
GRP
ACKR1
CCL21
CLDN6
ACTA2
TAGLN
COL1A1
ATI
ATII
ciliated (Cil)
goblet (Gob)
club
basal (Bas)
progenitors (Prog)
PNEC
vascular endothelial (Vas)
lymphatic endothelial (Lym)
smooth muscle (SM)
fibroblasts (Fib)

Figure S3. **Quality-control of the scRNA-seq data obtained from enriched epithelial cells. A**. Gating strategy employed for FACS enrichment of viable epithelial cells from 3 cryopreserved lung samples of normal donors. Percentage of viable (SyTOX^{-}) epithelial cells (EPCAM^{+}) are indicated in each plot from donors HLD11, HLD15, and HLD20. **B**. Re-analysis of each of the sorted EPCAM^{+} populations before processing for scRNA-seq. **C**. Violin plots showing the expression levels (Y-axis) of established markers (indicated above of each plot) for the definition of the identified cell clusters (X-axis). Color legend used for each cell type is indicated on the bottom right. PNEC: pulmonary neuroendocrine cells.

Table S1. Reagents and materials used for collection, processing, and cryopreservation of lung tissue.

Reagent	Product	Company	Cat no	Final concentration
**Tissue transport:** **CO_2-i^{+++}**	CO_2 independent medium	Thermo Fisher Scientific	18045054	Base medium
	Albumin fraction V, protease-free (BSA)	Carl Roth	T844.3	1%
	Penicillin/streptomycin (PS)	Fisher Scientific	15140122	1%
	Amphoterin B	Fisher Scientific	15290026	1%
**Tissue wash:** **HBSS^{++++}**	HBSS without Ca^{2+}, Mg^{2+}, and phenol red	Fisher Scientific	14175053	Base buffer
	UltraPure™ 0.5M EDTA, pH 8.0	Thermo Fisher Scientific	15575020	2 mM
	Albumin fraction V, protease-free (BSA)	Carl Roth	T844.3	1%
	Penicillin/streptomycin (PS)	Fisher Scientific	15140122	1%
	Amphoterin B	Fisher Scientific	15290026	1%
**Freezing containers**	Mr. Frosty	Thermo Fisher Scientific	5100-0001	
	Cryo-tubes 2.0mL	Sarstedt	72.380	
**Parenchyma and tumor freezing**	DMEM, high glucose, GlutaMAX™	Thermo Fisher Scientific	31966047	Base medium
	Fetal Bovine Serum (FBS)	Gibco	10270106	20%
	Dimethylsulfoxide (DMSO)	Carl Roth	A994.1	10%
**Airway & vessel freezing**	Cryostor	Sigma-Aldrich	C2874	
**Tissue fixation**	10% neutral buffered formalin	Sigma-Aldrich	HT501128	10%

Table S2. Steps and reagents used for tissue and bronchospheres paraffin embedding performed at Morphisto (Morphisto GmbH, Frankfurt, steps 1-29). Infiltration and embedding (steps 1-13) were performed using the Leica TP1050 infiltration system and Leica EG 1140 H paraffin embedding station. H&E staining (steps 14-29). Rehydrating and antigen retrieval for immunofluorescence studies of bronchospheres were performed at BioMed X (30-39).

Step	Solution	Reference	Company	Time (min:sec)	Vacuum	Temperature (°C)
1	70% Ethanol	12089	Morphisto	60:00	Yes	35
2	80% Ethanol	11579	Morphisto	60:00	Yes	35
3	90% Ethanol	11616	Morphisto	60:00	Yes	35
4	90% Ethanol	11616	Morphisto	60:00	Yes	35
5	96% Ethanol	11470	Morphisto	150:00	Yes	35
6	96% Ethanol	11470	Morphisto	150:00	Yes	35
7	100% Isopropanol	11365	Morphisto	120:00	Yes	35
8	100% Isopropanol	11365	Morphisto	120:00	Yes	35
9	Xylene	11070	Morphisto	120:00	Yes	40
10	Xylene	11070	Morphisto	120:00	Yes	40
11	Xylene:Paraffin (1:1)	11070/ 17932A2,5	Morphisto/ Engelbrecht	180:00	Yes	50
12	Paraffin	17932A2,5	Engelbrecht	180:00	Yes	58
13	Paraffin	17932A2,5	Engelbrecht	300:00	Yes	58
14	Xylene	11070	Morphisto	05:00	No	RT
15	Xylene	11070	Morphisto	05:00	No	RT
16	96% Ethanol	11470	Morphisto	05:00	No	RT
17	80% Ethanol	11579	Morphisto	05:00	No	RT
18	70% Ethanol	12089	Morphisto	05:00	No	RT
19	Distilled water	—	—	01:30	No	RT
20	Hematoxylin (acidic acc. to Mayer)	10231	Morphisto	10:00	No	RT
21	Distilled water	—	—	00:30	No	RT
22	Running tap water	—	—	05:00	No	RT
23	Eosin (1%, aqueous, pH 6)	10177	Morphisto	10:00	No	RT
24	Running tap water	—	—	04:00	No	RT
25	96% Ethanol	11470	Morphisto	01:30	No	RT
26	96% Ethanol	11470	Morphisto	02:00	No	RT
27	100% Isopropanol	11365	Morphisto	05:00	No	RT
28	Xylene	11070	Morphisto	05:00	No	RT
29	Xylene	11070	Morphisto	05:00	No	RT

30	Xylol (isomere)	9713.3	Carl Roth GmbH	10:00	No	RT
31	Xylol (isomere)	9713.3	Carl Roth GmbH	10:00	No	RT
32	100% Ethanol	LC86574	NeoLab Migge GmbH	10:00	No	RT
33	100% Ethanol	LC86574	NeoLab Migge GmbH	10:00	No	RT
34	95% Ethanol	LC86574	NeoLab Migge GmbH	05:00	No	RT
35	70% Ethanol	LC86574	NeoLab Migge GmbH	05:00	No	RT
36	50% Ethanol	LC86574	NeoLab Migge GmbH	05:00	No	RT
37	Distilled water	—	—	02:00	No	RT
38	PBS 1X	14190-094	Fisher Scientific	10:00	No	RT
39	Citrate buffer pH 6.0	ab93678	Abcam	20:00	No	100

Table S3. Reagents used for in vitro culture of explant-derived human lung fibroblasts.

Reagent	Product	Company	Cat no	Final concentration
**Explant wash**	**HBSS**$^{++++}$ (Supplementary table 1)			
**HLF-culture: DMEM**$^{+++}$	DMEM, high glucose, GlutaMAX™	Thermo Fisher Scientific	31966047	Base medium
	Fetal Bovine Serum (FBS)	Gibco	10270106	2%
	Penicillin/streptomycin (PS)	Fisher Scientific	15140122	1%
**Subculture**	Trypsin 0.05% EDTA	Thermo Fisher Scientific	25300054	
**Cell cryo-preservation**	DMEM, high glucose, GlutaMAX™	Thermo Fisher Scientific	31966047	Base medium
	Fetal Bovine Serum (FBS)	Gibco	10270106	20%
	Dimethylsulfoxide (DMSO)	Carl Roth	A994.1	10%

Table S4. Reagents used for basal cell isolation, in vitro culture (2D and 3D) and pre-embedding (3D).

Reagent	Product	Company	Cat no	Final concentration
**Soak buffer**	JMEM	Sigma-Aldrich	M8028	Base medium
	DTT	Carl Roth GmbH + Co KG	6908.3	0.5 µg/mL
	Dnase I	ProSpec-Tany TechnoGene	enz-417	1 µg/mL
**Wash buffer**	CO2-independent medium	ThermoFisher Scientific	18045-054	Base medium
	Nystatin	Sigma-Aldrich	N1638	100 U/mL
	Amphotericin B	Sigma-Aldrich	A2942	1.25 µg/mL
	Gentamicin	Sigma-Aldrich	G1397	50 µg/mL
	L-Glutamine, 200 mM	ThermoFisher Scientific	25030024	2 mM
**2D culture: PneumaCult Ex+++**	PneumaCult-Ex	STEMCELL Technologies	#05008	Base medium + 50X Supplement
	Penicillin/Streptomycin (PS)	Fisher Scientific	15140-122	1%
	Hydrocortisone	STEMCELL Technologies	#07926	96 ng/mL
	Heparin	STEMCELL Technologies	#07980	0.08 µg/mL
**Subculture**	Reagent Pack (Trypsin, HBSS, and TNS)	Lonza	CC-5034	
**3D culture: BEGM***	BEGM, + Single Quots Supplement Pack (CC-3171 + CC-4175, without gentamicin, triiodothy-ronine and retinoic acid)	Lonza	CC-3170	Base medium, 50 %
	DMEM (1X) + GlutaMAX-I	Thermo Fisher Scientific	31966-021	Base medium, 50%
	Retinoic acid (RA)	Sigma-Aldrich	R2625	100 nM
	Matrigel® Growth Factor Reduced (GFR) Basement Membrane Matrix	Corning	354230	- 5% in base medium<br>- 25% for well bottom
	ROCK inhibitor, Y-27632, 2HCl	Adooq Bioscience	129830-38-2	10 µM, at seeding only
**96-well plate**	Ultra-Low Attachment 96 Well, Round Bottom,Polystyrene	Costar	7007	
**Histogel**	Histogel	Thermo Scientific	HG-4000-012	

Table S5. Reagents used for the isolation and in vitro culture of distal-lung epithelial cells from parenchyma and tumor epithelial cells from cryopreserved squamous cell carcinomas.

Reagent	Product	Company	Cat no	Final concentration
**Tissue wash**	**HBSS^{++++}** (Supplementary table 1)			
**Tissue dissociation**	**CO_2-i^{+++}** (Supplementary table 1)			
	Human tumor dissociation kit	Miltenyi Biotec	130-095-929	
	ROCK inhibitor, Y-27632 2HCl	Adooq Bioscience	129830-38-2	10 μM
	DNase I	ProSpec-Tany TechnoGene	enz-417	100 μg/mL
	Gentle MACS dissociator	Miltenyi Biotec	130-093-235	
	Gentle MACS™ C tubes	Miltenyi Biotec	130-093-237	
	MACSmix™ tube rotator	Miltenyi Biotec	130-090-753	
	Fetal Bovine Serum (FBS)	Gibco	10270106	20%
	ACK lysis buffer	Thermo Fisher Scientific	A10492-01	
	100, 70 and 40 μm Falcon cell strainers	Neolab Migge	352360, 352350 and 352340	
**Epithelial culture**	SAGM Bullet Kit	Lonza Cologne GmbH	CC-3118 (CC-3119 & CC-4124)	Base medium
	Fetal Bovine Serum (FBS)	Gibco	10270106	1%
**Subculture**	Reagent Pack (Trypsin, HBSS, and TNS)	Lonza Cologne GmbH	CC-5034	

Table S6. Reagents used for flow cytometry.

Reagent	Product	Company	Cat no	Final concentration
**Blocking**	**HBSS^{++++}** (Supplementary table 1)			
	Human TruStain FcX™	BioLegend	422302	5 μL/10^6 cells in 0,1mL HBSS^{+++}
**Staining**	Anti-human CD326 (EPCAM) PE	Affymetrix eBioscience	12-9326-42	Recommended by supplier
	APC/Cy7 anti-human CD45	BioLegend	304014	Recommended by supplier
	CD45-Bv605	BD BioScience	564047	Recommended by supplier
	SyTOX blue viable dye	Thermo Fisher Scientific	S34857	Recommended by supplier
**Sort**	Falcon® 5mL round bottom polystyrene tube, with cell strainer snap cap	Neolab Migge	RN003621	
	**CO_2-i^{+++}** (Supplementary table 1)			
	FACS Aria IIu sorter 605 85μm – 3-laser, 9-color (5-3-2)	BD Bioscience		
	BD FACSDiva CS&T and accudrop beads	BD Bioscience	655050, 345249	

Table S7. Reagents used for immunofluorescence.

Reagent	Product	Company	Cat no	Final concentration
**Fixing**	PFA	Sigma-Aldrich	HT501128	4%
**Permeabilizing**	Triton X-100	Carl Roth	3051.3	0.3 %
**Blocking buffer**	BSA	Carl Roth	T844.3	5 %
	Normal Donkey Serum	Abcam	ab166643	2 %
	PBS	Fisher Scientific	14190-094	1X
**Incubation buffer**	BSA	Carl Roth	T844.3	1 %
	Normal Donkey Serum	Abcam	ab166643	2 %
	PBS	Fisher Scientific	14190-094	1X
**Staining (dilution in incubation buffer)**	VIM	Santa Cruz Biotechnology	sc-7557	1:200
	Alpha-SMA	Abcam	ab7817	1:100
	EPCAM	Cell Signaling	2929S	1:100
	KRT5	Abcam	ab52635	1:200
	TP63	Abcam	Ab124762	1:200
	MUC5AC	Abcam	ab212636	1:200
	FOXJ1	Sigma-Aldrich	HPA005714	1:50
	DAPI	Sigma-Aldrich	10236276001	1:5000
	ProLong Gold Antifade	Thermo Fisher Scientific	P36931	
	Donkey anti-mouse Alexa Fluor 568	Thermo Fisher Scientific	A10037	1:500
	Donkey anti-mouse Alexa Fluor 488	Thermo Fisher Scientific	A21202	1:500
	Donkey anti-goat Alexa Fluor 568	Thermo Fisher Scientific	A11057	1:500
	Donkey anti-goat Alexa Fluor 488	Thermo Fisher Scientific	A11055	1:500
	Donkey anti-rabbit Alexa Fluor 568	Thermo Fisher Scientific	A10042	1:500
	Donkey anti-rabbit Alexa Fluor 488	Thermo Fisher Scientific	A21206	1:500
	Donkey anti-rat Alexa Fluor 488	Thermo Fisher Scientific	A21208	1:500
**96-well imaging plate**	Imaging Plate 96 CG	zell-kontakt	5242-20	

Table S8. Reagents used for RNA isolation, RNA-seq and scRNA-seq.

Reagent	Product	Company	Cat no
**RNA isolation and quality control for RNA-seq**	RNeasy plus micro kit	Qiagen	74034
	RNase-Free DNase Set	Qiagen	79254
	Nuclease-Free Water (not DEPC-Treated)	Thermo Fisher Scientific	AM9937
	Qubit RNA HS assay kit	Thermo Fisher Scientific	Q32852
	Bioanalyzer 2100, model G2939A	Agilent	
	Agilent RNA 6000 Pico Kit	Agilent	5067-1513
	RNA ladder	Agilent	5067-1535
**scRNA-seq**	Chromium Single Cell A Chip Kit	10X Genomics	PN-120236
	Chromium Single Cell 3’ Library & Gel Bead Kit v2	10X Genomics	PN-120237
	Chromium i7 Multiplex Kit	10X Genomics	PN-120262

Table S9. Quality check of the RNA-seq data obtained from human lung fibroblasts derived from fresh (F) or cryopreserved (C) explants of 3 different donors (HLD1, 2, and 5) and 2 to 5 different areas of the lung tissue (R1-5).

Donor	Approach	Area	Sample name	Sequenced reads	Uniquely aligned reads	%	Reads aligned to exons	%
**HLD1**	Fresh	4	HLD1_F_R1	30,655,789	27,400,813	89.4	25,150,775	82.0
	Cryo	4	HLD1_C_R1	37,770,587	33,382,465	88.4	30,411,359	80.5
	Fresh	5	HLD1_F_R2	35,396,579	31,772,663	89.8	29,236,358	82.6
	Cryo	5	HLD1_C_R2	40,477,613	36,065,383	89.1	33,154,970	81.9
**HLD2**	Fresh	2	HLD2_F_R1	31,455,941	27,801,847	88.4	25,514,487	81.1
	Cryo	2	HLD2_C_R1	30,050,300	25,810,178	85.9	23,605,981	78.6
	Fresh	3	HLD2_F_R2	37,205,699	32,620,650	87.7	29,849,145	80.2
	Cryo	3	HLD2_C_R2	37,409,704	33,027,955	88.3	30,228,535	80.8
	Fresh	4	HLD2_F_R3	36,110,948	31,227,019	86.5	28,549,550	79.1
	Cryo	4	HLD2_C_R3	32,252,123	28,643,217	88.8	26,337,207	81.7
	Fresh	5	HLD2_F_R4	31,686,342	28,365,401	89.5	25,950,908	81.9
	Cryo	5	HLD2_C_R4	32,959,132	28,728,778	87.2	26,390,291	80.1
	Fresh	6	HLD2_F_R5	30,405,946	27,061,545	89.0	24,832,174	81.7
	Cryo	6	HLD2_C_R5	31,090,529	27,642,658	88.9	25,498,984	82.0
**HLD5**	Fresh	4	HLD5_F_R1	36,279,667	32,429,479	89.4	29,793,253	82.1
	Cryo	4	HLD5_C_R1	41,507,431	36,771,181	88.6	33,701,348	81.2
	Fresh	6	HLD5_F_R2	31,316,509	27,514,820	87.9	25,288,532	80.8
	Cryo	6	HLD5_C_R2	29,608,859	23,756,602	80.2	21,763,076	73.5

Table S10. Comparison of the quality check (QC) reports (10xqc.com) used for evaluating scRNA-seq from the 3 cryopreserved donors presented here (Cryo BX) to 11 fresh and 36 frozen publicly-available human samples (Chemistry Description= Single Cell 3' v2, scRNA-seq method= 10x Genomics 3'mRNA v2, Transcriptome= GRCh38).

QC metrics	Cryo BX (n= 3)	Fresh (n= 11)	Frozen (n= 36)	QC cut-off
Number of Reads	120,722,975	226,862,216	56,786,976	
Estimated Number of Cells	3,404	3,705	1,653	
Mean Reads per Cell	34,958	132,527	38,079	
Median Genes per Cell	1,026	993	282	
Total Genes Detected	21,026	18,323	16,134	
Median UMI Counts per Cell	3,170	3,749	548	
%Valid Barcodes	97	98	98	>75%
%Sequencing Saturation	77	90	86	>80%
%Q30 Bases in Barcode	95	98	98	>90%
%Q30 Bases in RNA Read	77	80	72	>70%
%Q30 Bases in Sample Index	90	92	95	>90%
%Q30 Bases in UMI	94	98	98	>90%
%Reads Mapped Confidently to Transcriptome	53	62	49	>30%
%Fraction Reads in Cells	93	76	60	High

Table S11. Characteristics of the donors used for viability assays (HLD2, 6, 8, and 10), fibroblast profiling (HLD1, 2 and 5), and scRNA-seq (HLD11, 15, and 20). *na*: not analyzed. ESI: emphysema score index, SCC: squamous cell carcinoma, ADC: adenocarcinoma

Patient	Gender	Age	Packs year	FEV_1 (%)	FEV_1 / FVC (%)	ESI	Tumor
**HLD1**	f	64	50	78.7	69.2	0	SCC
**HLD2**	m	82	50	73.1	78.2	na	SCC
**HLD5**	m	62	50	114.6	80.1	0	SCC
**HLD6**	m	60	50	66.3	61.4	na	SCC
**HLD8**	m	56	20	67.4	84.1	na	ADC
**HLD10**	f	74	28	115.1	87.7	na	ADC
**HLD11**	m	76	20	113.1	78.3	2	SCC
**HLD15**	m	67	15	108.4	79.3	1	SCC
**HLD20**	f	69	60	115.7	75.5	1	SCC
